# *interleukin-11* induces and maintains progenitors of different cell lineages during *Xenopus* tadpole tail regeneration

**DOI:** 10.1038/s41467-017-00594-5

**Published:** 2017-09-08

**Authors:** Hiroshi Tsujioka, Takekazu Kunieda, Yuki Katou, Katsuhiko Shirahige, Taro Fukazawa, Takeo Kubo

**Affiliations:** 1 Department of Biological Sciences, Graduate School of Science, The University of Tokyo, 7-3-1, Hongo, Bunkyo-ku, Tokyo, 113-0033 Japan; 2 Insutitute of Molecular and Cellular Biosciences, the University of Tokyo, 1-1-1, Yayoi, Bunkyo-ku, Tokyo, 113-0032 Japan

## Abstract

Unlike mammals, *Xenopus laevis* tadpoles possess high ability to regenerate their lost organs. In amphibians, the main source of regenerated tissues is lineage-restricted tissue stem cells, but the mechanisms underlying induction, maintenance and differentiation of these stem/progenitor cells in the regenerating organs are poorly understood. We previously reported that *interleukin-11* (*il-11*) is highly expressed in the proliferating cells of regenerating *Xenopus* tadpole tails. Here, we show that *il-11* knockdown (KD) shortens the regenerated tail length, and the phenotype is rescued by forced-*il-11*-expression in the KD tadpoles. Moreover, marker genes for undifferentiated notochord, muscle, and sensory neurons are downregulated in the KD tadpoles, and the forced-*il-11*-expression in intact tadpole tails induces expression of these marker genes. Our findings demonstrate that *il-11* is necessary for organ regeneration, and suggest that IL-11 plays a key role in the induction and maintenance of undifferentiated progenitors across cell lineages during *Xenopus* tail regeneration.

## Abstract (web summary)

*Xenopus laevis* tadpoles have maintained their ability to regenerate various organs. Here, the authors show that *interleukin-11* is necessary for organ regeneration, by inducing and maintaining undifferentiated progenitors across cell lineages during *Xenopus* tail regeneration.

## Introduction

Some lower vertebrates, such as fish and amphibians, have a prominent ability to regenerate their lost organs compared to mammals^1^. *Xenopus laevis* tadpoles can regenerate their lost tails, including all tissues that comprise tails, such as the notochord, muscle, spinal cord and other tissues, after amputation, and are used as model animals for the study of vertebrate organ regeneration. In the regeneration of *X. laevis* tails^2^ or axolotl limbs^3^, the main sources of the regenerated organs are lineage-restricted tissue stem cells. Although the mechanisms underlying the synergistic induction, maintenance, and differentiation of these stem and/or progenitor blastema cells during organ regeneration are fundamental in organ regeneration, the detailed molecular mechanisms are not well understood. We previously reported that *interleukin-11* (*il-11*) is highly expressed in the proliferating cells in regenerating *X. laevis* tadpole tails^4^, raising the possibility that IL-11 plays a crucial role in *Xenopus* tadpole tail regeneration.

IL-11 is a member of IL-6 family, and its signalling cascade has been extensively studied in mammals^5^. IL-11 binds to both IL-11 receptor alpha (IL11RA) and IL-6 signal transducer (IL6ST, also known as GP130)^6, 7^, and transduces signals through IL6ST^8^. IL6ST is a receptor subunit common to all IL-6 family cytokines. Activated IL6ST phosphorylates signal transducer and activator of transcription (Stat) 1 and 3^9^, and phosphorylated Stat1 and Stat3 translocate to the nucleus to activate the transcription of target genes^10, 11^. IL-11 also activates the mitogen-activated protein/extracellular signal-regulated kinase kinase (MEK) pathway^12^, and the phosphatidylinositol-3 kinase (PI3K) pathway^13^.

Some members of the IL-6 family are involved in regulating the differentiation of stem/progenitor cells. For example, IL-6 treatment differentiates B lymphocytes to antibody-forming cells^14^. Leukaemia inhibitory factor (Lif) inhibits the differentiation of mouse embryonic stem cells^15, 16^. IL-11 treatment is reported to maintain the expression of undifferentiated markers in human embryonic stem cells^17^. *il-11* is also suggested to be involved in regeneration. *il-11* is reported to be expressed in the regenerating heart of zebrafish, and forced expression of a dominant negative form of Stat3 inhibits the proliferation of cardiomyocytes and heart regeneration^18^. Based on these findings, Fang et al. speculated that IL-11 is a candidate upstream molecule of the Stat3 pathway that is responsible for the proliferation of cardiomyocytes during regeneration. The precise role of *il-11* in regeneration of organs comprised of various tissues, however, is not clear.

Here, we produced *il-11* knockeddown (KD) tadpoles using the CRISPR/Cas9 system to show that *il-11* is necessary for tail regeneration in *X. laevis* tadpoles. In addition, the shortened regenerated tails, a phenotype of the *il-11* KD tadpoles, is rescued by forced expression of *il-11* at the amputated tail stumps. In the amputated tail stumps of the *il-11* KD tadpoles, marker genes for undifferentiated notochord, muscle and sensory neurons are downregulated compared to control tadpoles. Furthermore, forced expression of *il-11* in the intact tadpole tails induces expression of the markers for undifferentiated cells. Our results strongly suggest that IL-11 plays a key role in the induction and maintenance of undifferentiated progenitor cells across cell lineages during *Xenopus* tadpole tail regeneration.

## Results

### *il-11* is induced after tail amputation

**Fig. 1:**
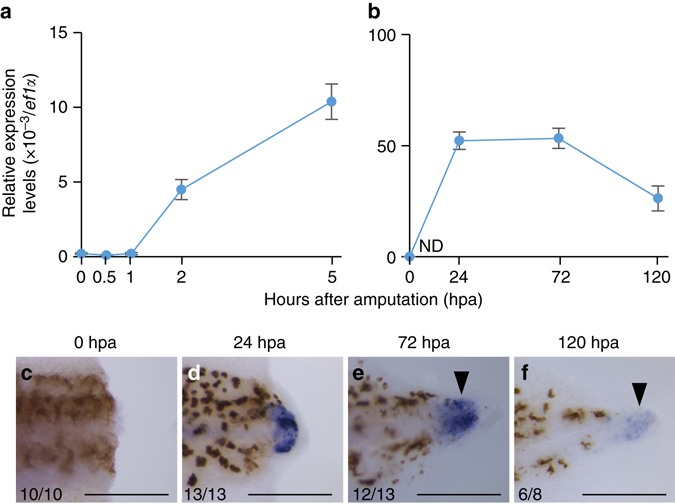
*il-11* is expressed at the tip of blastema during tail regeneration. **a**, **b** Expression levels of *il-11* were measured by qRT-PCR using RNA extracted from ~20 tadpoles. Tail stump tissues cut at the level of 0.5 mm anterior from the amputated plane were used. *Vertical axes* represent relative expression levels of *il-11* normalised by those of *ef1α*. Mean ± s.e.m. *n* = 4. ND: not detected. **c**–**f** WISH was performed using tadpoles fixed at 0 **c**, 24 **d**, 72 **e**, or 120 hpa **f**. Anterior is to the *left* and dorsal is *up*. *Blue*/*purple colour* represents signals for *il-11* expression. *Brown pigments* are melanophores of the tadpoles. *Scale bars*: 500 μm. Numbers at the *bottom corner* indicate the total ratio of tadpoles showing the corresponding expression pattern from at least two batches. Note that *il-11* was expressed at the blastema tip at 72 and 120 hpa (*black arrowheads*)

First, we examined the correlation between the cellular processes and *il-11* expression in regenerating *Xenopus* tadpole tails. Quantitative reverse transcription-polymerase chain reaction (qRT-PCR) of *il-11* mRNA in the amputated tail stumps of tadpoles collected at 0, 0.5, 1, 2 and 5 h post amputation (hpa) showed that the *il-11* expression began at 2 hpa (Fig. 1a), suggesting that *il-11* is related to early events immediately after tail amputation. We then examined *il-11* expression levels in later phases after amputation (24, 72 and 120 hpa). *il-11* expression was maintained for at least 120 hpa (Fig. 1b), suggesting that *il-11* is also related to some late events after tail amputation.

Whole-mount in situ hybridisation (WISH; Fig. 1c–f, Supplementary Fig. 1) revealed no significant *il-11* expression at 0 hpa at the amputated tail stumps, coinciding with the qRT-PCR results. Significant *il-11* expression was detected at the outer edge of the notochord and spinal cord near the amputation plane at 5 and 24 hpa, indicating that *il-11* expression was restricted to the amputation plane at the early phase of tail regeneration. At 72 and 120 hpa, *il-11* expression was detected at the tip of the regenerating tails, where the tail blastema cells are located (Fig. 1e, f). We previously observed that proliferating tail blastema cells begin to accumulate at the tip of the regenerating tails 3 days post amputation (dpa)^4^. Induction of *il-11* expression at the amputated tail stumps in the early phases (5 and 24 hpa) and its suspension at the tip of regenerating tails in the later phases (>3 dpa) led us to hypothesise that *il-11* is involved in the induction and/or maintenance of tail blastema cells, which are undifferentiated progenitor cells of various tissues in the tail.

To investigate whether *il-11* is selectively expressed among IL-6 family members, we performed qRT-PCR for *il-6* and *lif*, which encode IL-6 family members important for the differentiation or maintenance of stem/progenitor cells^14–16^. Expression of these two genes was modestly induced (at most, less than approximately threefold) after tail amputation compared to the expression of *il-11* (Supplementary Fig. 2a–d), suggesting that *il-11* has a specific function in tail regeneration among IL-6 family members.

To investigate whether IL-11 signalling is actually activated, we visualised activation of Stat3, which is the major signalling molecule and transcription factor downstream of IL-11^10, 11^, by immunohistochemistry using an antibody against phosphorylated Stat3. We found that phosphorylated Stat3 co-localised with the nuclei in notochord bud, spinal cord ampulla and epithelium in blastema of 72-hpa tadpoles (Supplementary Fig. 3). Although Stat3 is activated by several upstream ligands, including IL-6 and Lif, expression of these two ligands was not prominently elevated compared to that of *il-11* after tail amputation, as mentioned above, and thus it is plausible that the observed activation of Stat3 is mainly due to the expression of IL-11, and that IL-11 signalling is activated in various cell types in blastema.

To investigate whether IL-11–responsive cells are induced after tail amputation, we performed qRT-PCR for *il11ra* and *il6st*, which are genes for receptors of IL-11^6, 7^. Expression of these genes was modestly induced (at most, less than approximately threefold; Supplementary Fig. 2e–h) compared to the expression of *il-11*, suggesting that the IL-11–responsive cells constitutively reside in intact tails and the number of the cells remains largely unaltered during the regeneration process.

### *il-11* is necessary for tail regeneration

**Fig. 2:**
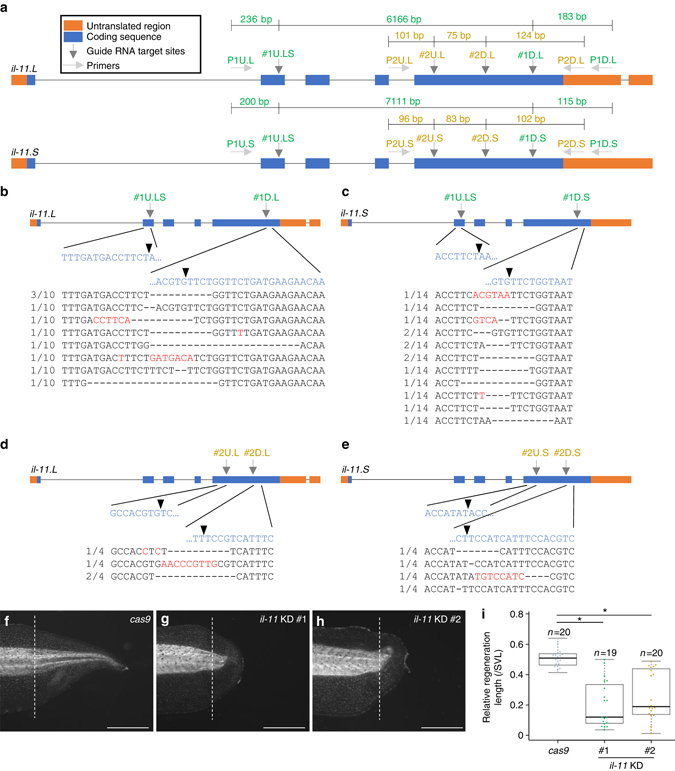
Knockdown of *il-11* in tadpoles. **a** Schematic drawing of genomic structure of *il-11*. Bars: *introns*, *orange boxes*: untranslated regions, *blue boxes*: coding sequences, *dark grey* *vertical arrows*: gRNA target sequences, *light grey horizontal arrows*: primers. **b**–**e**, Genomic sequences of tadpoles injected with *cas9* mRNA and gRNAs. Genomic DNA was extracted from all body of *il-11* KD #1 tailbud embryos **b**, **c** or tail of *il-11* KD #2 tadpoles **d**, **e**, and sequences corresponding to *il-11.L* **b**, **d** or *il-11.S* **c**, **e** were PCR-amplified using P1U.L/P1D.L **b**, P1U.S/P1D.S **c**, P2U.L/P2D.L **d**, or P2U.S/P2D.S **e** primers. The PCR product of the expected length corresponding to deletion of the region between two gRNA target sites was sequenced. *Blue characters*: reference genomic sequences nearby gRNA target sites, *red characters*: mismatches from reference genomic sequences, *bars*: gaps, *arrowheads*: predicted cleavage sites. Numbers at the *left corner* indicates ratio of clones containing the sequence to total clones sequenced. **f**–**i**, Representative images of tails of control *cas9* mRNA-injected **f**, *il-11* KD #1 **g**, or *il-11* KD #2 **h** tadpoles at 7 dpa, and the tail regeneration length **i** are shown. Dark-field images **f**–**h** are shown. Anterior is to the *left*, dorsal is *up*. *White broken lines* indicate amputated plane. *Scale bars*: 1 mm. Regeneration length was normalised by snout to vent length (SVL). *Box plots* are inserted in the panels. *Bars* in the boxes represent median, upper and lower limits of the boxes represent the first and third quartiles, and whiskers represent maximum and minimum values. *P < 0.05, Dunnett’s test

We next examined whether *il-11* is necessary for tail regeneration by knocking-down *il-11* using the CRISPR/Cas9 system. Because *X. laevis* has a long generation time of more than 1 year, we attempted to analyse the effect of *il-11* KD in F_0_ tadpoles. To efficiently knockdown *il-11* in F_0_ tadpoles, we designed guide RNAs (gRNAs) targeting two regions each in two *il-11* homoeologues of allotetraploid *X. laevis*, *il-11.L* and *il-11.S* (Fig. 2a), which is expected to cause a large deletion of the nucleotide sequences sandwiched by the two gRNA target sites, in addition to the frame shift that occurs at each of the two gRNA target sites of each gene. We co-injected these gRNAs (#1U.LS, #1D.L and #1D.S) and *cas9* mRNA into fertilised eggs, and kept the hatched tadpoles till approximately stage 40–42. We then amputated the tails of F_0_ tadpoles (*il-11* KD #1 tadpoles) at 6 days post fertilisation (dpf), and measured the regenerated tail length at 7 dpa (13 dpf). To confirm that the observed phenotype was not due to an off-target effect, we also created another group of KD tadpoles (*il-11* KD #2 tadpoles) using another gRNA set targeting other sequences in *il-11* (Fig. 2a), and compared the regenerated tail length of *il-11* KD #1, #2 and control *cas9* mRNA-injected tadpoles (*cas9* tadpoles). We detected a loss of genomic regions between the two gRNA target sites of *il-11.L* and *il-11.S* using PCR amplification of the interval regions (Fig. 2b–e), indicating that *il-11* genes could be edited by this approach. The ratio of normally developed *il-11* KD tadpoles was not prominently lower than that of *cas9* tadpoles (Supplementary Table 1), suggesting that *il-11* KD does not affect normal development under these conditions. The ratio of normally grown *il-11* KD tadpoles after tail amputation was almost the same as that of *cas9* tadpoles (Supplementary Table 1), suggesting that *il-11* does not have a crucial role in wound healing or in immune responses after tail amputation. When we measured relative regenerated tail length normalised by snout to vent length (Supplementary Fig. 4f and g), we found that the regenerated tail length was significantly shorter in both *il-11* KD #1 and #2 tadpoles compared to *cas9* tadpoles (*P* < 0.05, Dunnett’s test, Fig. 2f–i, Supplementary Fig. 4h), suggesting that *il-11* is necessary for tail regeneration. Deletion of genomic regions between the two gRNA target sites of *il-11.L* and *il-11.S* was detected in the tail stump tissues of most of these tadpoles (Supplementary Fig. 4a–e), suggesting that the function of IL-11 was lost in at least some cells at the amputated tail stumps of most of these *il-11* KD tadpoles.

**Fig. 3:**
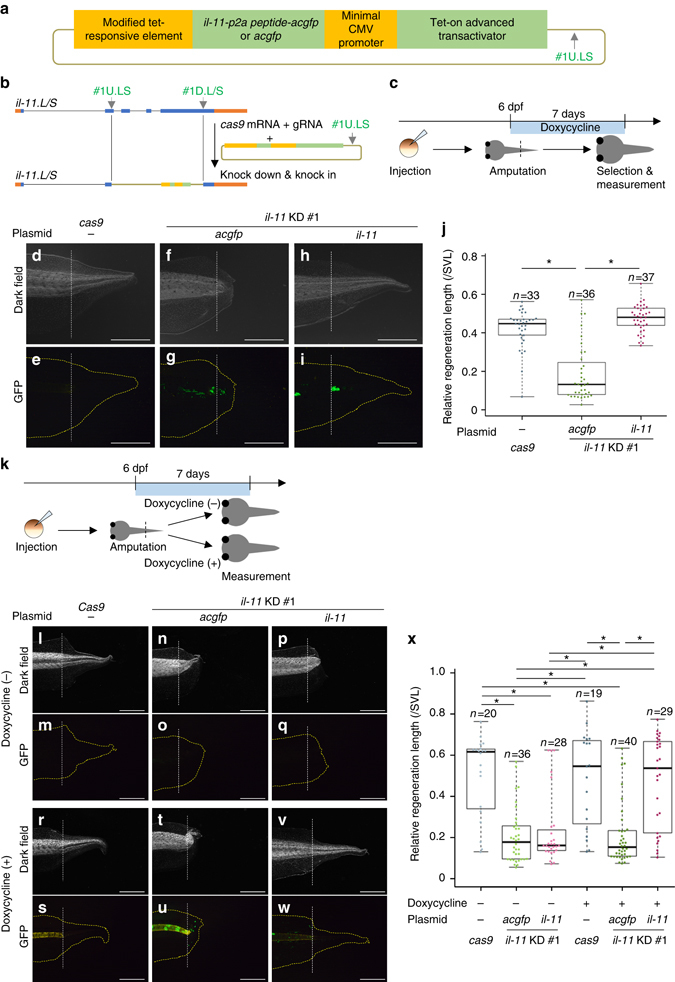
Rescue experiment of the phenotype of *il-11* KD tadpoles. **a** Schematic drawing of a rescue construct. *Yellow boxes* represent cis-regulatory elements, and *green boxes* represent coding sequences. gRNA #1U.LS target site was inserted in the construct. In the control construct, *acgfp* was inserted instead of *il-11-p2a peptide-acgfp*. **b** Schematic drawing of knock-in and knockdown. *Bars*: introns, *orange boxes*: untranslated regions, *blue boxes*: coding sequences, *dark grey arrows*: gRNA target sequences. **c** Schematic drawing of a rescue experiment. **d**–**j** Representative images of tails of control *cas9* mRNA-injected **d**, **e**, *il-11* knockeddown *acgfp*-expressing **f**, **g**, or *il-11* knockeddown *il-11*–expressing **h**, **i** tadpoles, and their regeneration length **j** are shown. **k**, Schematic drawing of a rescue experiment using tadpoles treated with or without doxycycline. **l-x** Representative images of tails of untreated *cas9* mRNA-injected **l**, **m**, *il-11* knockeddown *acgfp*-expressing **n**, **o**, or *il-11* knockeddown *il-11*–expressing **p**, **q** tadpoles, or doxycycline-treated *cas9* mRNA-injected **r**, **s**, *il-11* knockeddown *acgfp*-expressing **t**, **u**, or *il-11* knockeddown *il-11*–expressing **v**, **w** tadpoles and their regeneration length **x** are shown. Dark-field images **d**, **f**, **h**, **l**, **n**, **p**, **r**, **t**, **v** and GFP2-filtered images **e**, **g**, **i**, **m**, **o**, **q**, **s**, **u**, **w** are shown. Regeneration length was normalised by snout to vent length (SVL). *Box plots* are inserted in the panels. *Bars* in the boxes represent median, upper and lower limits of the boxes represent the first and third quartiles, and *whiskers* represent maximum and minimum values. *Scale bars*: 1 mm. Anterior is to the *left*, dorsal is *up*. *Yellow broken lines* indicate outline of the tails. *White broken lines* indicate amputated plane. *P < 0.05, Tukey−Kramer’s test

To confirm that the shortened regeneration length was not due to a non-specific effect of chromosomal disruption, which might have been caused by genome editing, we performed a rescue experiment of the phenotype of *il-11* KD tadpoles. For this, an *il-11* rescue construct that expresses IL-11 with the expression marker AcGFP upon doxycycline treatment by the tet-on system (Fig. 3a), or a control construct that expresses AcGFP alone, was co-injected with *cas9* mRNA and gRNAs targeting *il-11* into a one-cell stage embryo. In the *il-11* rescue and control *acgfp*-expressing constructs, the target sequence of gRNA #1U.LS was inserted. Some constructs might be knocked into the *il-11* gene locus by homology-independent double-strand break repair^19^ when the genomic *il-11* locus and the target sequence on the rescue construct were cleaved concurrently by the CRISPR/Cas9 system (Fig. 3b). In addition to the free constructs remaining in the cells of the tadpole tail at the time of tail amputation, the knocked-in constructs would facilitate the expression of *il-11* by stably inserting into the genome. The injected tadpoles were kept till approximately stage 40–41, then their tails were amputated at 6 dpf following doxycycline treatment, and their regenerated tail length was measured at 7 dpa (13 dpf; Fig. 3c, Supplementary Fig. 5a, b). To select tadpoles in which the constructs were successfully introduced, normally grown tadpoles with GFP signal at the amputation plane were selected under a fluorescence stereomicroscope for measurement of the regeneration length of tadpoles with forced *acgfp* or *il-11* expression (Supplementary Table 2). In the *cas9* group, all normally grown tadpoles were selected, because *cas9* tadpoles do not show GFP signals (Supplementary Table 2). Although the relative regenerated tail length of *il-11* KD *acgfp*-expressing construct-introduced tadpoles was significantly shorter compared to *cas9* mRNA-injected tadpoles, the tail shortening was almost completely rescued in *il-11*-rescued tadpoles (*P* < 0.05, Tukey−Kramer’s test, Fig. 3d–j, Supplementary Fig. 5c), suggesting that *il-11* is necessary for tail regeneration.

**Fig. 4:**
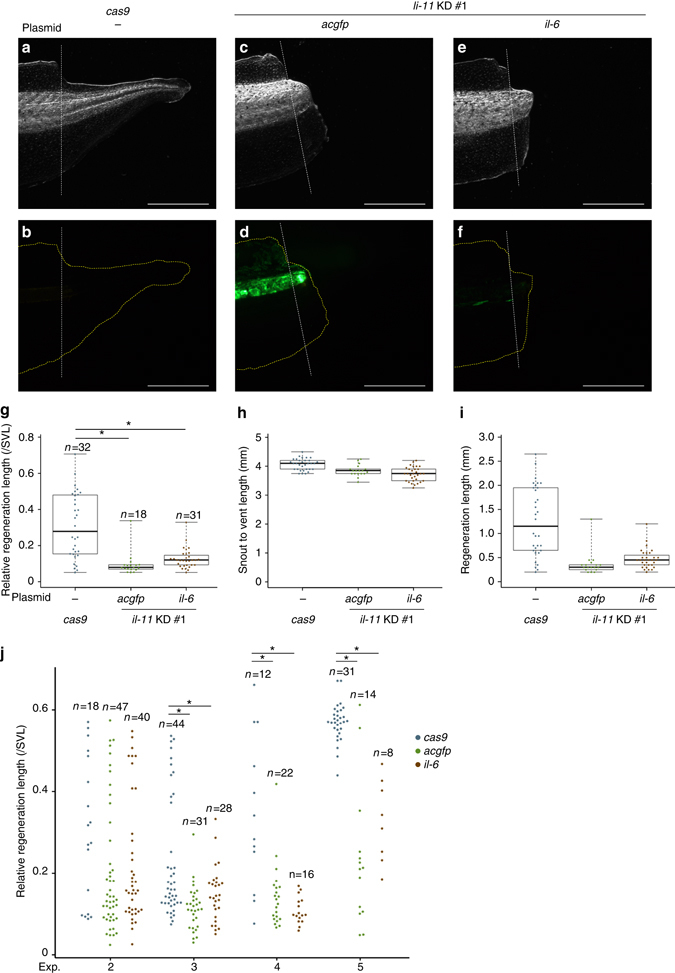
*il-6* does not rescue the shortened regeneration length of *il-11* KD tadpoles. **a**–**f** Representative images of tails of control *cas9* mRNA-injected **a**, **b**, *il-11* knockeddown *acgfp*-expressing **c**, **d**, or *il-11* knockeddown *il-6*–expressing **e**, **f** tadpoles, and their regeneration length **g** are shown. Dark-field images **a**, **c**, **e** and GFP2-filtered images **b**, **d**, **f** are shown. Regeneration length was normalised by snout to vent length (SVL). **h**, **i**, SVL **h** and regeneration length **i** used for calculation in **g** are shown. **j** Biological replicates using different batches of tadpoles are shown. Box plots are inserted in the panels. *Bars* in the boxes represent median, upper and lower limits of the boxes represent the first and third quartiles, and *whiskers* represent maximum and minimum values. *Scale bars*: 1 mm. Anterior is to the *left*, dorsal is *up*. *Yellow broken lines* indicate outline of the tails. *White broken lines* indicate amputated plane. *P < 0.05, Tukey−Kramer’s test

We also investigated whether the rescue effect is specific for *il-11* among the IL-6 family members by forcing the expression of *il-6* in *il-11* KD tadpoles. Normally grown tadpoles with GFP signal at the amputation plane were selected for measurement of the regeneration length (Supplementary Table 3). Regeneration length was almost the same in *il-11* KD tadpoles with forced expression of *il-6* compared to *il-11* KD tadpoles expressing *acgfp* (Fig. 4), suggesting that signalling pathways specific to *il-11* among IL-6 family members are necessary for tail regeneration.

To further confirm that the rescue effect of *il-11* is not due to the introduced construct itself, but to the expression of *il-11*, we compared the regeneration length of doxycycline-treated and untreated tadpoles. Injected tadpoles were divided into two groups after tail amputation at 6 dpf: the first group was maintained in 0.1× Steinberg’s solution and the second group was maintained in 0.1× Steinberg’s solution with 50 μg/ml of doxycycline, and regeneration length was measured at 7 dpa (Fig. 3k). The ratio of normally grown tadpoles after tail amputation was not prominently different between the doxycycline-treated and untreated groups (Supplementary Table 4), suggesting that doxycycline treatment did not affect tadpole viability under these conditions. The regeneration length of all normally developed tadpoles was measured and normalised by the snout to vent length (Supplementary Fig. 5d, e). GFP signals were very weak in untreated *acgfp-* and *il-11*−expressing construct-introduced tadpoles compared to their doxycycline-treated counterparts (Fig. 3o, q, u, w), suggesting that the expression of AcGFP and IL-11 is doxycycline-dependent. In the untreated group, regeneration length was significantly shorter both in *acgfp-* and *il-11*−expressing construct-introduced tadpoles compared to *cas9* tadpoles (Fig. 3l–q, x, Supplementary Fig. 5f). In the doxycycline-treated group, however, regeneration length was significantly shorter in *acgfp*-expressing construct-introduced tadpoles compared to *il-11*−expressing construct-introduced and *cas9* tadpoles, and the regeneration length of *il-11*–expressing construct-introduced tadpoles was almost the same as that of *cas9* tadpoles (*P* < 0.05, Tukey−Kramer’s test, Fig. 3r–w, x, Supplementary Fig. 5f), clearly indicating that *il-11* is necessary for tail regeneration.

### *il-11* induces and maintains progenitors of several tissues

Next, to analyse the role of *il-11* in tail regeneration, we compared gene expression profiles between the amputated tail stumps of *il-11* KD and *cas9* tadpoles. When we compared the morphology of regenerating tails of *il-11* KD and *cas9* tadpoles every day after tail amputation, they were almost the same until 2 dpa. At 3 dpa, however, *cas9* tadpoles showed prominent growth of the regenerated tail, whereas *il-11* KD tadpoles showed only moderate growth (Supplementary Fig. 6), suggesting that some regenerative processes present between 2 and 3 dpa were disrupted in *il-11* KD tadpoles. Therefore, we compared gene expression profiles in tail stump tissues of *il-11* KD #1 and *cas9* tadpoles at 2 dpa by RNA-sequencing.

**Fig. 5:**
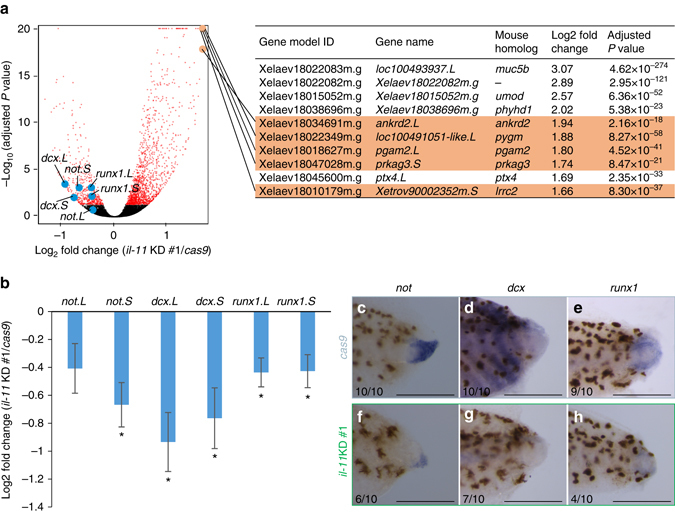
*il-11* induces or maintains tissue progenitor cells across cell lineages during tail regeneration. **a** Gene expression profile of *il-11* KD #1 and *cas9* tadpoles. *Left panel*: volcano plot. Fold-change of expression level of *il-11* KD #1 tadpoles over control tadpoles measured by RNA-sequencing are plotted on horizontal axis in log_2_ scale, and adjusted *P*-values are plotted on vertical axis in –log_10_ scale. Genes with significant fold-changes (adjusted *P* < 0.05) are plotted in *red*. Points beyond the range are plotted on the edge of the graph. Representative undifferentiated markers are plotted in *blue*. *Right panel*: top 10 genes which showed highest upregulation in *il-11* KD #1 are listed. Mouse homologues of genes indicated in *orange* are reported to be selectively expressed in skeletal muscle in mice (Supplementary Fig. 7). These genes correspond to *orange* plots in the *left panel*. **b** Fold-change of expression levels of representative undifferentiated markers measured by RNA-sequencing. Mean ± s.e.m. *n* = 3. *adjusted *P* < 0.05, Wald test. **c**–**h**, Localisation of undifferentiated markers. Representative WISH images for *not* **c**, **f**, *dcx* **d**, **g**, or *runx1* **e**, **h** in control **c**–**e** or *il-11* KD #1 tadpoles **f**–**h** are shown. Anterior is to the *left*, dorsal is *up*. *Blue*/*purple colour* represents signals for the genes. *Brown pigments* are melanophores of the tadpoles. *Scale bars*: 500 μm. Numbers at the bottom corner indicate the total ratio of tadpoles showing the corresponding expression pattern from two batches

We found that among top 10 genes which showed highest upregulation in *il-11* KD #1 tadpoles, five genes are reported to be selectively expressed in skeletal muscle in mouse (Fig. 5a and Supplementary Fig. 7), suggesting that ratio of mature tissues were relatively high in tail stump tissues of *il-11* KD tadpoles compared to *cas9* tadpoles. This prompted us to hypothesise that ratio of undifferentiated cells were relatively high in tail stump tissues of *cas9* tadpoles compared to *il-11* KD tadpoles. Then we found that the expression levels of several undifferentiated cell markers were affected by *il-11* knockdown (Fig. 5a, b). In *il-11* KD #1 tadpoles compared to *cas9* tadpoles, we observed a tendency toward or significant (adjusted *P* < 0.05, Wald test) downregulation of *notochord homeobox* (*not*), an early notochord marker in *Xenopus* ^20, 21^; *doublecortin* (*dcx*), a marker for a subpopulation of muscle progenitor cells in mouse^22, 23^; and *runt related transcription factor 1* (*runx1*), a marker of progenitor cells of Rohon-Beard sensory neurons^24^, which are a subpopulation of primary sensory neurons located in the dorsal spinal cord that mediate the response to touch in larval stages of lower vertebrates.

When we performed WISH (Fig. 5c–h, Supplementary Fig. 8), *not* was expressed at the tip of the notochord bud, *dcx* was expressed in the muscle near the amputation plane, and *runx1* was expressed in the spinal cord ampulla and ventral side of the blastema. The ratio of tadpoles with strong expression of the genes tended to be higher in the *cas9* group (*not*: 10/10, *dcx*: 10/10, and *runx1*: 9/10) compared to the *il-11* KD #1 and #2 groups (*not*: 4/10 and 1/10; *dcx*: 3/10 and 3/10; *runx1*: 6/10 and 7/10). These results suggest that induction and/or maintenance of the progenitor cells of the notochord, muscle, or Rohon-Beard sensory neurons are impaired in *il-11* KD tadpoles.

We also performed section ISH for the undifferentiated marker genes using doxycycline-treated *cas9* mRNA-injected, *acgfp-* or *il-11*−expressing *il-11* KD tadpoles fixed at 2 dpa. We detected signals for *not* and *dcx*. Expression of *not* was detected in the notochord bud, and expression of *dcx* was detected in the muscle and spinal cord (Supplementary Fig. 9). The ratio of tadpoles with strong expression in the notochord bud (*not*) or muscle (*dcx*) tended to be higher in *cas9* (*not*: 6/6, *dcx*: 11/11) and *il-11*-rescued (*not*: 6/6. *dcx*: 11/11) tadpoles compared to control *acgfp-*expressing *il-11* KD tadpoles (*not*: 2/6, *dcx*: 3/11), further suggesting that induction and maintenance of undifferentiated cells are *il-11–*dependent. The expression of *dcx* in the spinal cord seems to be *il-11* independent, because signals for *dcx* in the spinal cord were observed in tadpoles in all groups.

### *il-11* can induce progenitor cells in intact tail

**Fig. 6:**
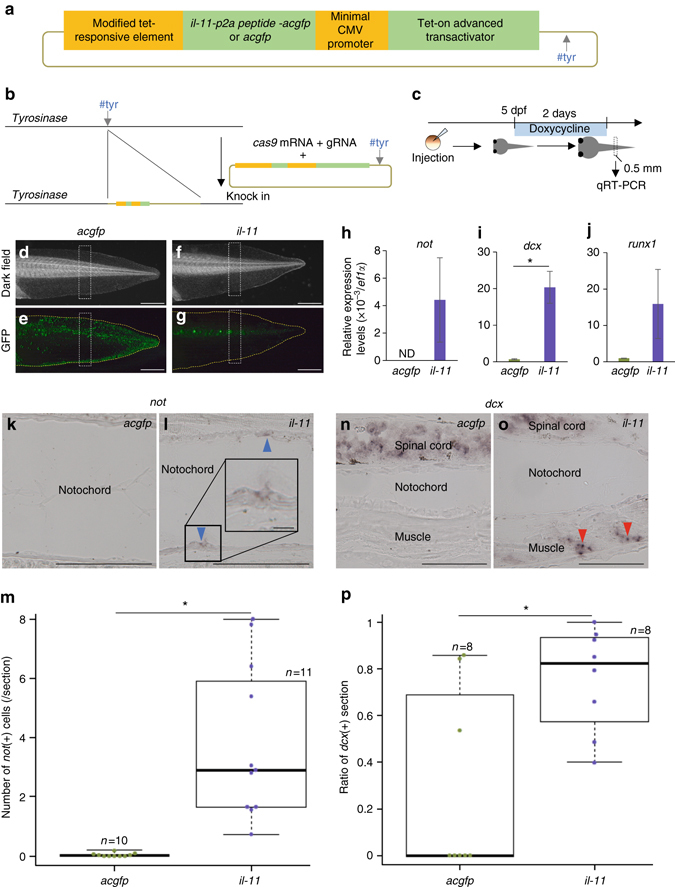
*il-11* is sufficient for induction of progenitor cells across cell lineages in intact tadpole tail. **a** Schematic drawing of a construct for forced expression of *il-11*. *Yellow boxes* represent cis-regulatory elements, and *green boxes* represent coding sequences. gRNA #tyr target site was inserted in the construct. In the control construct, *acgfp* was inserted instead of *il-11-p2a peptide-acgfp*. **b** Schematic drawing of knock-in. *Dark grey arrows*: gRNA #tyr target sequences. **c** Schematic drawing of a gain of function experiment. **d**–**g**, Representative images of tadpoles used in this experiment. *acgfp*-**d**, **e** or *il-11*-**f**, **g** expressing tails 2 days after doxycycline treatment are shown. Tail tissues indicated in *white broken lines* from approximately 10 tadpoles were used for qRT-PCR. Dark-field images **d**, **f** and GFP2-filtered images **e**, **g** are shown. *Scale bars*: 1 mm. Anterior is to the *left*, dorsal is *up*. *Yellow broken lines* indicate outline of the tails. **h**–**j**, Expression levels of *not* **h**, *dcx* **i**, or *runx1* **j** in tails expressing *acgfp* or *il-11* were measured by qRT-PCR. *Vertical axes* represent relative expression levels normalised by those of *ef1α*. Mean ± s.e.m. *n* = 3. **k**, **l**, **n**, **o**, Representative ISH images for *not* **k**, **l** or *dcx* **n**, **o** in *acgfp* **k**, **n** or *il-11* **l**, **o**-expressing intact tadpole tails 2 days after doxycycline treatment are shown. Several *not*-expressing cells in the notochord sheath (*blue arrowheads*) and *dcx*-expressing muscle (*red arrowheads*) were detected in *il-11*−expressing tails. **m** The number of *not*-expressing cells in the notochord sheath in all sections containing notochord was counted. **p** The ratio of sections containing *dcx*-expressing muscle in all sections containing muscle was calculated. Box plots are inserted in the panels. *Bars* in the boxes represent median, upper and lower limits of the boxes represent the first and third quartiles, and *whiskers* represent maximum and minimum values. *Scale bars*: 100 μm except inset in (l) (10 μm). Anterior is to the *left*, dorsal is *up*. *Magenta*/*purple* colour represents signals for the genes. *Inset*: magnified view of boxed area. *P < 0.05, Student’s *t*-test. ND: not detected

Finally we examined whether expression of *il-11* is sufficient for the induction and/or maintenance of the progenitor cells even in intact tails. For this, we co-injected *acgfp*- or *il-11*–expressing vector with *cas9* mRNA and gRNA targeting the *tyrosinase* locus, which is the gene responsible for albinism and unnecessary for tadpole survival, into the one-cell stage embryo to facilitate the expression of *il-11* by construct knocked into the *tyr* locus, as well as free plasmid retained in the cells of the tadpole tails. Then, the injected tadpoles at 5 dpf (approximately stage 40–42) were treated with doxycycline for 2 days, and the expression levels of the undifferentiated cell marker genes in their tails were compared (Fig. 6a–g). Although *not* expression was scarcely detected, or detected at very low levels in *acgfp*-expressing tails, it was detected in *il-11*–expressing tails (Fig. 6h, Supplementary Fig. 10a). *dcx* was significantly upregulated in *il-11*–expressing tails compared to *acgfp*-expressing tails (*P* < 0.05, Student’s *t*-test, Fig. 6i, Supplementary Fig. 10b). The expression level of *runx1* tended to be higher in *il-11*–expressing tails than in *acgfp*-expressing tails (Fig. 6j, Supplementary Fig. 10c).

To investigate whether the number of cells expressing undifferentiated marker genes is increased by forced expression of *il-11*, we performed section ISH. We detected signals for *not* and *dcx*. *not* signals were detected in cells in the notochord sheath of *il-11*–expressing tadpoles, whereas few signals were detected in *acgfp*-expressing cells (Fig. 6k–m, Supplementary Fig. 11a). *dcx* signals were detected in the spinal cord and muscle (Fig. 6n, o, Supplementary Fig. 11b). The ratio of muscle expressing *dcx* was higher in *il-11*–expressing tadpoles compared to *acgfp*-expressing tadpoles (Fig. 6p), suggesting that IL-11 is sufficient to induce and maintain progenitor cells even in intact tails. Notably, the fact that the genes upregulated in *il-11*–expressing tails were undifferentiated cell markers of different tissues suggests that induction of IL-11 alone is sufficient to induce progenitor cells derived from multiple tissues in intact tail, without additional factors that are induced by tail amputation.

## Discussion

In the present study, we first analysed the expression of *il-11*. *il-11* was expressed in the spinal cord and notochord at the amputation plane early after tail amputation, and continued to be expressed in the tip of the spinal cord ampulla and notochord bud in later phases of regeneration (Supplementary Fig. 1g, h). These regions correspond to the area where *runx1-* or *not-*expressing cells reside (Supplementary Figs. 8k, m, o, q, 9a–c). Therefore, *il-11* might induce and maintain these progenitor cells in an autocrine/paracrine manner.

Next, we investigated whether *il-11* is necessary for tail regeneration by creating *il-11* KD/rescue tadpoles. In *il-11*-rescued tadpoles, GFP signals were sparse (Fig. 3i, w). It is important to note that IL-11 is cleaved from AcGFP at the P2A peptide, and therefore GFP signals reflect the localisation of not IL-11 itself, but of *il-11*−expressing cells. One possible explanation for how the sparse expression of *il-11* could rescue the shortened regeneration length is that IL-11 was very strongly expressed in the source cells and secreted everywhere in the tail at a high concentration, and IL-11–responsive cells that express IL-11 receptors at the tip of the blastema received the IL-11 signal. The result that the forced expression of *il-11* in the intact tail prominently upregulated expression of undifferentiated marker genes (Fig. 6) supports the idea that the concentration of IL-11 is sufficient for undifferentiated cells to respond even though the GFP signal was sparse. Because we did not observe additional tails or mass in the tails with forced *il-11* expression, it is possible that additional signals at the tip of the blastema are needed for prolonged survival or continued proliferation of the progenitor cells.

We then analysed the role of *il-11* in tail regeneration. We found that marker genes of progenitor cells in different cell lineages were downregulated in *il-11* KD tadpoles (Fig. 5), and upregulated in tadpoles with forced *il-11* expression (Fig. 6). Notably, these marker genes were not exclusively expressed in a single cell population. *dcx* was expressed in the spinal cord (Fig. 6n, o, Supplementary Figs. 8l, n, p, 9e–h, 11b) as well as in muscle. *dcx* is expressed in neuronal precursor cells in the developing mouse brain^25^. Therefore, it is possible that these cells are neural progenitors, although they might be *il-11* independent, because the signal for *dcx* in the spinal cord was not prominently downregulated in *il-11* KD tadpoles (Supplementary Figs. 8n, p, 9h), or upregulated in tadpoles with forced expression of *il-11* (Fig. 6n, o, Supplementary Fig. 11b). *runx1* was expressed in the ventral region of the amputated tail stumps in addition to the spinal cord (Supplementary Fig. 8m, o, q). *runx1* is expressed in hemogenic endothelium in mice^26^. The dorsal aorta is located at the ventral side of the *Xenopus* tadpole tail. Therefore, *runx1* might also be expressed in the postulated hemogenic endothelium at the amputated plane in *Xenopus*. Further studies, such as lineage tracing of cells expressing the markers or transplantation analysis of the progenitors will more strongly support the idea that the cells induced and maintained by *il-11* are actually progenitor cells of various cell lineages.

Although previous studies reported that *il-11* is expressed in regenerating heart in zebrafish^18^, and that IL-11 maintains expression of undifferentiated markers in human embryonic stem cells^17^, our findings are the first to indicate that IL-11 plays a key role in the induction and maintenance of the progenitor cells of various tail tissues. Downstream of IL-11, it is probable that some intracellular signals, which have been investigated in mammals^5^, function to maintain a broad range of stem/progenitor cells in an undifferentiated state. Analysing the molecular mechanisms that link tail amputation to *il-11* induction, such as the identification of a regeneration-specific enhancer element, which is suggested in zebrafish^27^, will unveil regeneration-specific molecular and cellular processes that start after amputation in organ regeneration.

## Methods

Statistical tests were not used to pre-determine sample size. The experiments were not randomised or blinded. Normally developed tadpoles were used in the experiments.

### Animals and tail amputation

Animals were obtained and treated as described previously^4^. In brief, tadpoles were obtained by mating wild-type *Xenopus laevis*, and maintained in 0.2% salt water at 20 °C until they were used. Tadpole tails were amputated at 5 days post fertilisation (dpf) (Nieuwkoop and Faber stage^28^ 39–42) after anaesthetisation with 0.02% MS-222 (Sigma-Aldrich, St. Louis, MO). We followed the Guidelines for Proper Conduct of Animal Experiments of Science Council of Japan. The protocol in this study was approved by the Committee on the Ethics of Animal Experiments of the Graduate School of Science at the University of Tokyo.

### qRT-PCR

qRT-PCR was performed essentially as described previously^4^ with minor modifications. In brief, total RNA was extracted using RNeasy mini kit (Qiagen, Germany), and reverse transcribed using PrimeScript RT reagent Kit with gDNA Eraser (Perfect Real Time; TaKaRa, Japan). Targets were amplified using SYBR premix ExTaq II (Tli RNaseH plus; TaKaRa) and a LightCycler 480 Real-time PCR System (Roche Diagnostics, Switzerland). The amounts of the transcripts were normalised with those of *elongation factor-1 alpha* (*ef1a)*. The forward and reverse primers used for amplification of each gene were as follows: *ef1α*; 5′-GGAACGGTGACAACATGC-3′ and 5′-AGGCAGACGGAGAGGCTTA-3′, *il-11*; 5′-TCCTGAAGCTAAGCACTGACCT-3′ and 5′-TGAATTCCGTTAAATTCGTGGTCCA-3′, *il-6*; 5′-TTGTGGCCACTCACCTTGGT-3′ and 5′-GATGAGCTTCGCTAGGGCCA-3′, *lif*; 5′-AACAGCGATGCCATTGGACA-3′ and 5′-TGCTCCAGATGCCAGCTCTT-3′, *il11ra*; 5′-AATCACATCGCTGGCGTGTG-3′ and 5′-CATCCAGCAGCCGGAAACTG-3′, *il6st*; 5′-CCAGAAGCTCCACCTTCCAG-3′ and 5′-CCATTGGCTACGGAGTCACTT-3′, *not*; 5′-GAACAGACAGACCTGCCTCC-3′ and 5′-AGCGGTAGGGCATAGATGGG-3′, *dcx*; 5′-TGCCGATTCAGCCAATGGAA-3′ and 5′-TGCTTACGAAGGCTTCCTGG-3′, *runx1*; 5′-CAGCCCGCATCACCCAAATC-3′ and 5′-TCTGACCCTGAGGCTGAGGA-3′.

### WISH

WISH was performed as described previously^4^. In brief, whole body of the tadpoles were fixed with MEMFA (50 mM 3-(N-morpholino) propanesulfonic acid, pH 7.4, including 2 mM ethylene glycol-bis(2-aminoethylether)-N,N,N′,N′-tetraacetic acid, 1 mM MgSO_4_, 3.7% formaldehyde) fixative, and kept in methanol at −20 °C until they were stained. Then they were rehydrated, treated with 50 μg/ml of proteinase K for 10 min, treated with 0.5% of acetate anhydride in 0.1 M of triethanolamine, and fixed with 3.7% formaldehyde. The specimens were pre-hybridised in the hybridisation buffer (Denhardt’s solution (Wako, Japan) including 50% formamide, 0.75 M NaCl, 0.075 M trisodium citrate, 100 μg/ml heparin, 0.1% Tween 20, 0.1% 3-((3-Cholamidopropyl) dimethylammonio) propanesulfonate, and 1 mg/ml Ribonucleic acid from torula yeast Type VI (Sigma-Aldrich)) for 4–6 h at 60 °C, and hybridised with the probe solution (1 μg/ml of digoxigenin labelled RNA probe in hybridisation buffer) for 4 nights at 60 °C. They were treated with RNase solution (1% RNase Cocktail (Invitrogen, Waltham, MA) in 0.3 M NaCl and 0.03 M trisodium citrate), following incubation with alkaline phosphatase conjugated anti-digoxigenin antibody and chromogenic reaction with a nitro blue tetrazolium chloride/5-bromo-4-chloro-3-indolyl phosphate, toluidine salt solution. After staining, they were treated with 10% H_2_O_2_ in phosphate-buffered saline to bleach the melanophore of the tadpoles, although some of the melanophores remained in the specimens. Following staining, some of the specimens were embedded in 4% agar in phosphate-buffered saline, and sagittally sliced at 30-μm intervals using a vibratome VT1000S (Leica Biosystems, Germany).

### Immunohistochemistry

Immunohistochemistry was performed essentially as described previously^29^. In brief, tadpole tails were amputated at 5 dpf (stage 39–41) and fixed at 3 dpa (stage 47) with MEMFA fixative, embedded with 25% gelatin 15% sucrose, frozen, sagittally sliced at 10 μm thick, and incubated with anti-phospho-Stat3 (Tyr705) mouse antibody (Cell Signalling Technology, MA, #9138S) following incubation with Alexa Fluor 555-conjugated anti-mouse IgG goat antibody (Invitrogen, A-21424). Nuclei were counterstained with 10 μg/ml of Hoechst 33342 (Lonza, Switzerland).

### Knockdown experiment

gRNAs^30^ and *cas9* ^31^ mRNA were designed and synthesised essentially as described previously with minor modifications. In brief, DR274^30^ plasmid was digested with *Bsa* I-HF (New England Biolabs, MA), annealed with oligonucleotide, and they were ligated. Some of the gRNA template plasmids were assembled using a PCR-amplified DR274 fragment, an oligonucleotide, and an In-Fusion HD Cloning Kit (TaKaRa). After transformation and plasmid extraction, the plasmid was digested with *Dra* I (*Aha* III; TaKaRa), following PCR amplification and in vitro transcription using AmpliScribe T7-*Flash* Transcription Kit (Epicentre, WI). The gRNA was purified using RNeasy mini kit. The number of possible off-target sites of the obtained gRNAs was predicted by searching *Xenopus laevis* genome 9.1 (Xenbase^32, 33^) using Cas-OFFinder^34^ and possible gRNAs that recognised any sites other than the target genes in the genome were not used. gRNA target sequences were as follows: #1U.LS; GGTGCATAGCCATGTTTAGAAGG, #1D.L; GGACTATGCAACACGTGTTCTGG, #1D.S; GGACTTTGCAACACGTGTTCTGG, #2U.L; GCACCATATGCCACGTGTCACGG, #2U.S; GTTCCGTGACACGTGGTATATGG, #2D.L; GAGGTGGAAATGACGGAAAATGG, #2D.S; GACGTGGAAATGATGGAAGATGG. For *cas9* mRNA synthesis, pXT7-hcas9^31^ (China Zebrafish Resource Center, China, CZP2) was digested with *Xba* I (TaKaRa), following in vitro transcription using mMESSAGE mMACHINE T7 ULTRA Kit (Invitrogen). The obtained *cas9* mRNA was purified using RNeasy mini kit.

The injections were carried out essentially as described previously^29^ with the modifications. In brief, fertilised eggs obtained by artificial fertilisation were dejellied by incubation with 3% cysteine, following injection in Ficoll solution (2% Ficoll 400 in 10 mM NaCl, 0.2 mM KCl, 0.1 mM MgCl_2_, 0.2 mM CaCl_2_, 0.5 mM 2-(4-(2-Hydroxyethyl)-1-piperazinyl)ethanesulfonic acid, pH 7.5) using Nanoject II (Drummond Scientific Company, PA). We injected *cas9* mRNA (700 ng/μl) and gRNAs (20 ng/μl each, #1U.LS, #1D.L, and #1D.S in *il-11* KD #1, and #2U.L, #2U.S, #2D.L, and #2D.S in *il-11* KD #2) at a volume of 18.4 nl into single-cell stage embryos. Embryos were injected and kept at 12 °C until gastrulation began, and reared at 20 °C thereafter. The tails were amputated from the tadpoles at 6 dpf, and the tail regeneration length was measured at 7 dpa.

Genomic DNA was extracted using DNeasy Blood and Tissue kit (Qiagen) or by boiling tissues in 50 mM NaOH at 98 °C for 10 min followed by adding 1/10 volume of 1 M Tris-HCl (pH 7.5). Primers used for amplification of genomic regions around the gRNA target sites and annealing temperatures were as follows: P1U.L and P1D.L; 5′-TGACTCAGTACTGCGATATTTCCA-3′ and 5′-AGGTCCTAAAATGTCCCGGT-3′ (53 °C), P1U.S and P1D.S; 5′-TTTTGCTGGGACTTCGTTGC-3′ and 5′- ACCTCAGGCGTTTGATGCTA-3′ (58 °C), P2U.L and P2D.L; 5′-AGAGTTTCTGGATAATGGACCCC-3′ and 5′-ATATGTGCCCGTTCTGCAGT-3′ (60 °C), P2U.S and P2D.S; 5′-GGAGAGATAACTAAAAGCTAACACAA-3′ and 5′-ACATCAAGGATGGAGGTTTGTGA-3′ (60 °C). Primers used for amplification of positive control *ef1α* were the same as described in the qRT-PCR section. PCR products were cloned into pGEM-T easy vector (Promega, Madison, WI, USA) and sequenced.

The statistical significance of the difference in the relative regeneration length normalised by snout to vent length was assessed by Dunnett’s test or Student’s *t*-test with a cutoff of *P* < 0.05.

### Rescue experiment

The rescue construct was assembled using the In-Fusion HD Cloning Kit (TaKaRa). In brief, the *il-11* fragment was PCR-amplified using the *il-11.L* sequence (AB933563)^4^ cloned into pGEM-T easy vector (Promega). Fragments containing *acgfp*, or minimal CMV promoter and Tet-On Advanced transactivator were PCR-amplified using pAcGFP1-N1 (TaKaRa), or pTet-On Advanced (TaKaRa) as templates, respectively. Oligonucleotides for Gly-Ser-Gly-P2A peptide^35^ and the gRNA #1U.LS target sequence were as follows: 5′-GGAAGCGGAGCTACTAACTTCAGCCTGCTGAAGCAGGCTGGAGACGTGGAGGAGAACCCTGGACCT-3′ and 5′-AGGTCCAGGGTTCTCCTCCACGTCTCCAGCCTGCTTCAGCAGGCTGAAGTTAGTAGCTCCGCTTCC-3′, 5′-CAGCTCGACCAAGCTCCTTCTAAACATGGCTATGCACC-3′ and 5′-AAATCTCGCCAAGCTGGTGCATAGCCATGTTTAGAAGG-3′. These fragments and oligonucleotides were inserted into pTRE-Tight (TaKaRa). To prevent double-strand breaks, synonymous substitutions were introduced at the target sequence for #1U.LS and #1D.L in the *il-11.L* sequence of the rescue construct by PCR using mutated primers as follows: 5′- TGATGATTTGCTAAACATGGCTATGCAC-3′ and 5′- TTTAGCAAATCATCAAATTCCACTTTT-3′, 5′-TTGTTCTGATGAAGAACAAGTTGGGA-3′ and 5′-TCTTCATCAGAACAAGAACACGTGTT-3′.

For construction of the plasmid used for the forced expression of *il-6*, the *il-11* sequence of the *il-11* rescue construct was substituted by the coding sequence of *il-6.S* cloned into a pGEM-T easy vector using the In-Fusion system.

Injection was carried out as described in the knockdown experiment section except that 6 ng/μl of the rescue construct was added to the injection solution. Tails from injected tadpoles were amputated at 6 dpf, tadpoles were treated with 50 μg/ml doxycycline in 0.2% salt water as described previously^29^ for 7 days immediately after tail amputation, and tail regeneration length was measured. Only tadpoles showing green fluorescence near the amputation plane or a regenerated tail were used in the *acgfp*-expressing and *il-11* rescue groups. Tadpoles showing extremely high green fluorescence were excluded to prevent possible adverse effects of a high concentration of AcGFP. This criterion was not pre-established.

In experiments comparing the regeneration length of the untreated and doxycycline-treated groups, tadpoles were incubated in either 0.1× Steinberg’s solution (0.3 mM 2-(4-(2- hydroxyethyl)-1-piperazinyl)ethanesulfonic acid, pH 7.4, including 5.8 mM NaCl, 67 μM KCl, 34 μM Ca(NO_3_)_2_, 83 μM MgSO_4_) or 0.1× Steinberg’s solution with 50 μg/ml of doxycycline after tail amputation, and the regeneration length of all normally grown tadpoles were measured.

The statistical significance of the difference in the relative regeneration length normalised by snout to vent length was assessed by Tukey−Kramer’s test with a cutoff of *P* < 0.05.

### RNA-sequencing

Library construction, sequencing, and mapping were performed essentially as described previously^4^ with the minor modifications. In brief, tail stump tissues cut at the level 0.5 mm anterior from the amputated plane were collected at 2 dpa from ~20 *cas9* mRNA-injected or *il-11* KD #1 tadpoles from 3 different batches. Total RNA was extracted using RNeasy mini kit. cDNA library was produced using TruSeq Stranded Total RNA with Ribo-Zero Gold LT Sample Prep Kit (Illumina, CA). We produced a set of approximately 5 × 10^7^ single end reads (66 bp) from each cDNA library using Hiseq 2500 (Illumina). *X. laevis* genome 9.1 (Xla.v91.repeatMasked.fa; Xenbase^32, 33^) was used as a reference for mapping. The number of reads mapped to each gene was counted using HTSeq^36^ with a reference gene model (XL_9.1_v1.8.3.2.primaryTranscripts.gff3; Xenbase^32, 33^) and compared using DESeq2^37^. A volcano plot was created using ggplot2^38^. Gene expression levels of mouse homologues were obtained from BioGPS^39–41^ using a microarray data set of a previous study in mouse^42^.

### Forced expression in intact tail

The gRNA #tyr target sequence was as follows: 5′-GGCTCCATGTCTTCCGTCCAAGG-3′. Although an off-target sequence was found in the latest *Xenopus laevis* genome 9.1, most tadpoles survived after injection. Construction for forced expression of *il-11* was carried out as described in the rescue experiment section except that no mutation was introduced in *il-11* sequences, and the gRNA #tyr target sequence was introduced instead of #1U.LS using oligonucleotide as follows: 5′-CAGCTCGACCAAGCTGGCTCCATGTCTTCCGTCCAAGG-3′ and 5′-AAATCTCGCCAAGCTCCTTGGACGGAAGACATGGAGCC-3′

Injection and doxycycline treatment were carried out as described in the rescue experiment section except that concentration of gRNA #tyr was 40 ng/μl, and embryos were maintained at 20 °C after they developed to the 16-cell stage. Doxycycline treatment started at 5 dpf, and continued for 2 days following sampling for qRT-PCR or ISH. Tadpoles with GFP signals in the region approximately corresponding to the tail tissues used for qRT-PCR were used for the analyses.

### Tissue section ISH

ISH was performed essentially as described previously^43^. In brief, tadpoles were fixed with Bouin’s fixative, embedded in Paraplast (McCormick Scientific, St. Louis, MO). Eight micrometre-thick sagittal sections was prepared, and rehydrated. The slides were incubated with digoxigenin-labelled probe solution overnight, following incubation with alkaline phosphatase conjugated anti-digoxigenin antibody and chromogenic reaction with a nitro blue tetrazolium chloride/5-bromo-4-chloro-3-indolyl phosphate, toluidine salt solution.

For detection of *dcx*, tadpoles were fixed with MEMFA instead of Bouin’s fixative. Three probes targeting different regions of *dcx* were mixed and used to increase detection sensitivity.

### Data availability

RNA-sequencing data have been deposited in the DNA DataBank of Japan under accession code PRJDB5211.

We used already deposited GSE10246 data set^42^ in Gene Expression Omnibus of NCBI for expression analysis in mouse tissues.

High resolution supplementary figures are available at doi:10.6084/m9.figshare.5131867.

We declare that all data supporting the findings of this study are available within the article and its supplementary information files or from the corresponding author upon reasonable request.

## Electronic supplementary material

Peer Review File

Supplementary Information
