# Supplementary material for: interleukin-11 induces and maintains progenitors of different cell lineages during Xenopus tadpole tail regeneration
Source: Nat Commun. 2017 Sep 8;8:495. doi: 10.1038/s41467-017-00594-5 (PMC5591189; doi:10.1038/s41467-017-00594-5)
Supplement: Supplementary file 2 — Supplementary Information [file 41467_2017_594_MOESM2_ESM.pdf]

Description of Supplementary Files

File Name: Peer Review File

File Name: Supplementary Information
Description: Supplementary Figures, Supplementary Tables, Supplementary References.

Supplementary Figures

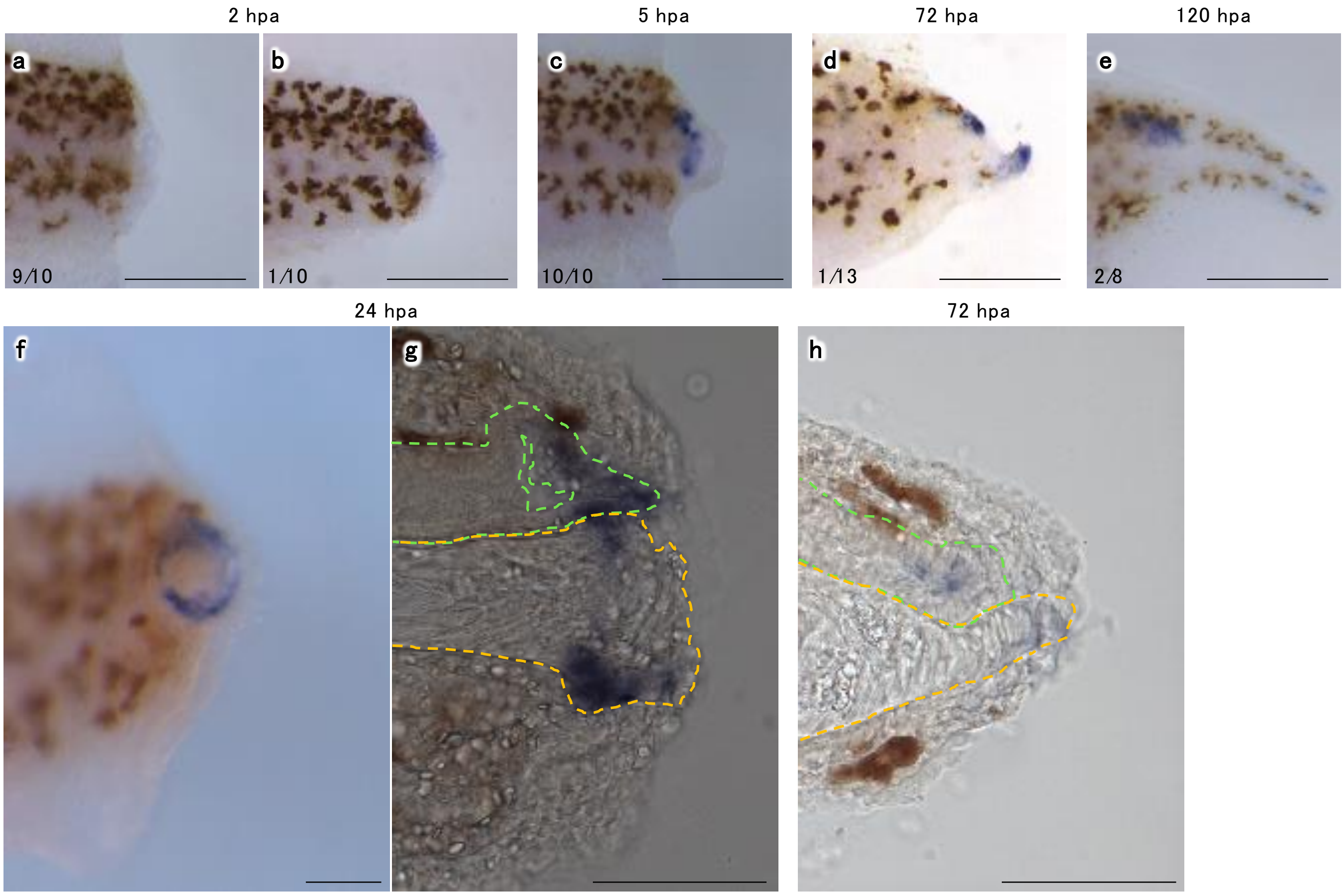

Supplementary Figure 1 Detailed localisation analysis of *il-11* after tail amputation

a-**e**, WISH images for *il-11* that are not shown in Fig. 1. Representative images of tadpoles fixed at 2 (a and b), 5 (c), 72 (d), or 120 hpa (e) are shown. **f**, Posterior view of a tadpole fixed at 24 hpa. **g**, **h**, Sagittal section of tadpoles fixed at 24 (g) or 72 (h) hpa. Anterior is to the left and dorsal is up in side view or sagittal section images. Dorsal is up in the posterior view. Blue/purple colour represents signals for *il-11* expression. Brown pigments are melanophores of the tadpoles. Scale bars: 500 µm (a-e) or 100 µm (f-h). Numbers at the bottom corner are the total ratio of tadpoles showing the corresponding expression pattern from at least 2 batches. Green broken lines indicate spinal cord and spinal cord ampulla, and yellow broken lines indicate notochord and notochord bud.

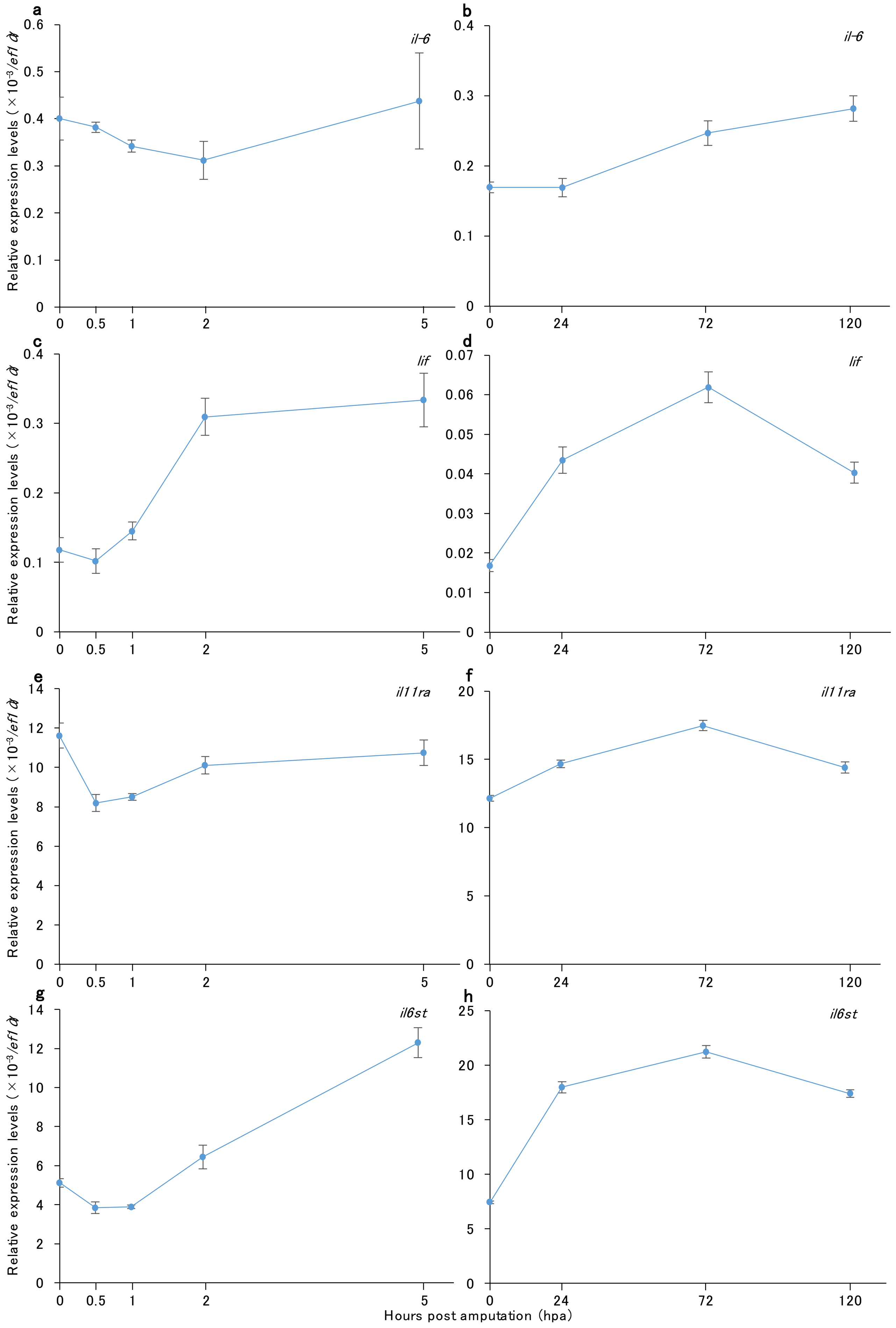

a
b
c
d
e
f
g
h
il-6
lif
il11ra
il6st
Relative expression levels (×10⁻³/ef1α)
Hours post amputation (hpa)
0
0.5
1
2
5
24
72
120

Supplementary Figure 2 Expression levels of members of the IL-11 family and receptors for IL-11

Expression levels of *il-6* (a, b), *lif* (c, d), *il11ra* (e, f), or *il6st* (g, h) were measured by qRT-PCR using RNA extracted from approximately 20 tadpoles. Tail stump tissues cut at the level of 0.5 mm anterior from the amputated plane were used. Vertical axes represent relative expression levels of the genes normalised by those of *ef1α*. Mean ± s.e.m. n=4.

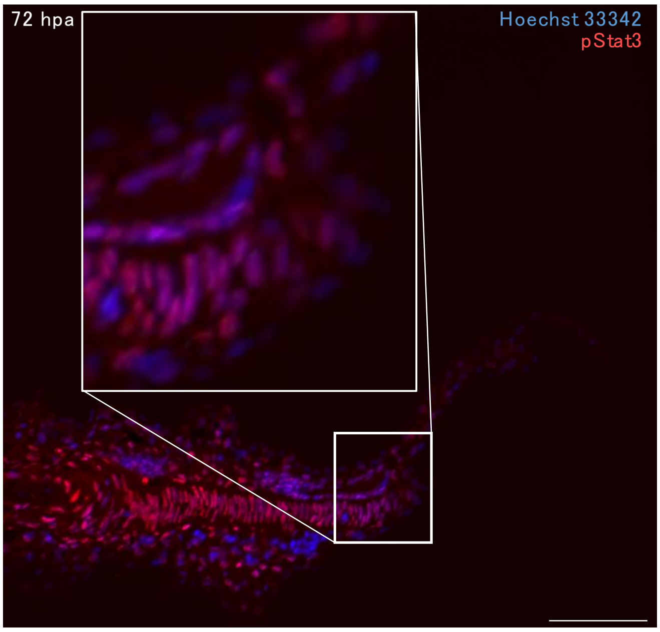

Supplementary Figure 3 Localisation of phosphorylated Stat3 in blastema

Tadpoles fixed at 72 hpa were sagittally sliced, and labelled using antibody against phosphorylated Stat3 (red). Nuclei were counterstained with Hoechst 33342 (blue). Anterior is to the left, dorsal is up. Inset: magnified view of boxed area. Scale bar: 100 μm.

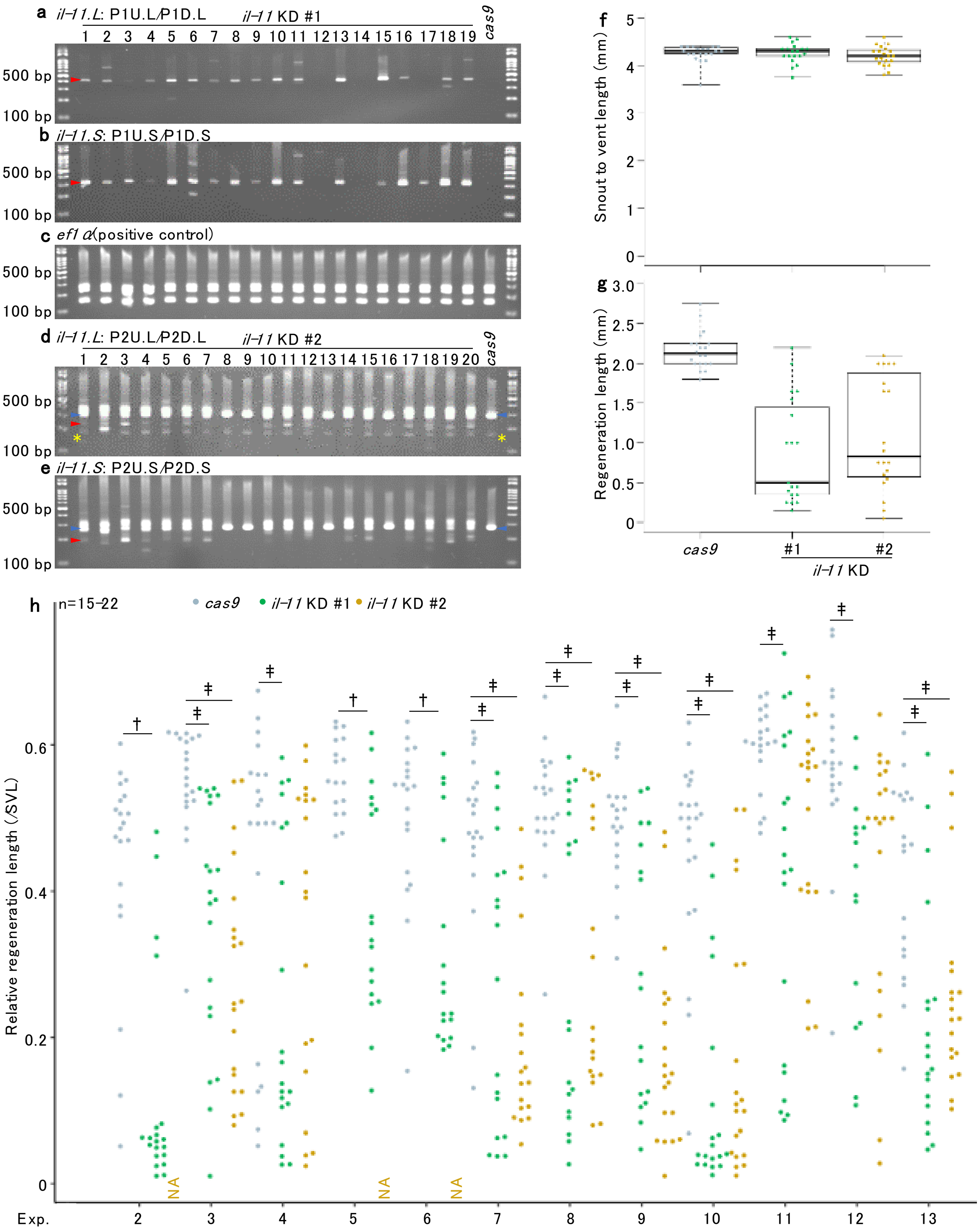

Supplementary Figure 4 Analyses of *il-11* knocked-down tadpoles

a-e, Genotyping of tadpoles used in Fig. 2i. Genomic DNA was extracted from tail stump tissues cut at the level 0.5 mm anterior from the amputated plane after measurement of regeneration length, and sequences corresponding to *il-11.L* (a, d), *il-11.S* (b, e), or positive control *ef1a* (c) were PCR-amplified. Individual identification numbers are indicated on top. Sizes of DNA markers were (from bottom to top) 100, 200, 300, 400, 500, 600, 700, 800, 900, 1000, 1500, and 3000 bp. If the genomic region between two gRNA target

sites is deleted, a band will be detected as indicated by the red arrowhead. Blue arrowheads correspond to wild-type sequences. Yellow asterisks: non-specific bands. **f**, **g**, Snout to vent length (SVL, f) and regeneration length (g) used for calculation in Fig. 2i are shown. **h**, Biological replicates for Fig. 2i are shown. Different batches of tadpoles were used in each experiment (Exp.). Box plots are inserted in the panels. Bars in the boxes represent median, upper and lower limits of the boxes represent the first and third quartiles, and whiskers represent maximum and minimum values. †$P < 0.05$, Student's *t*-test. ‡$P < 0.05$, Dunnett's test. NA: not analysed.

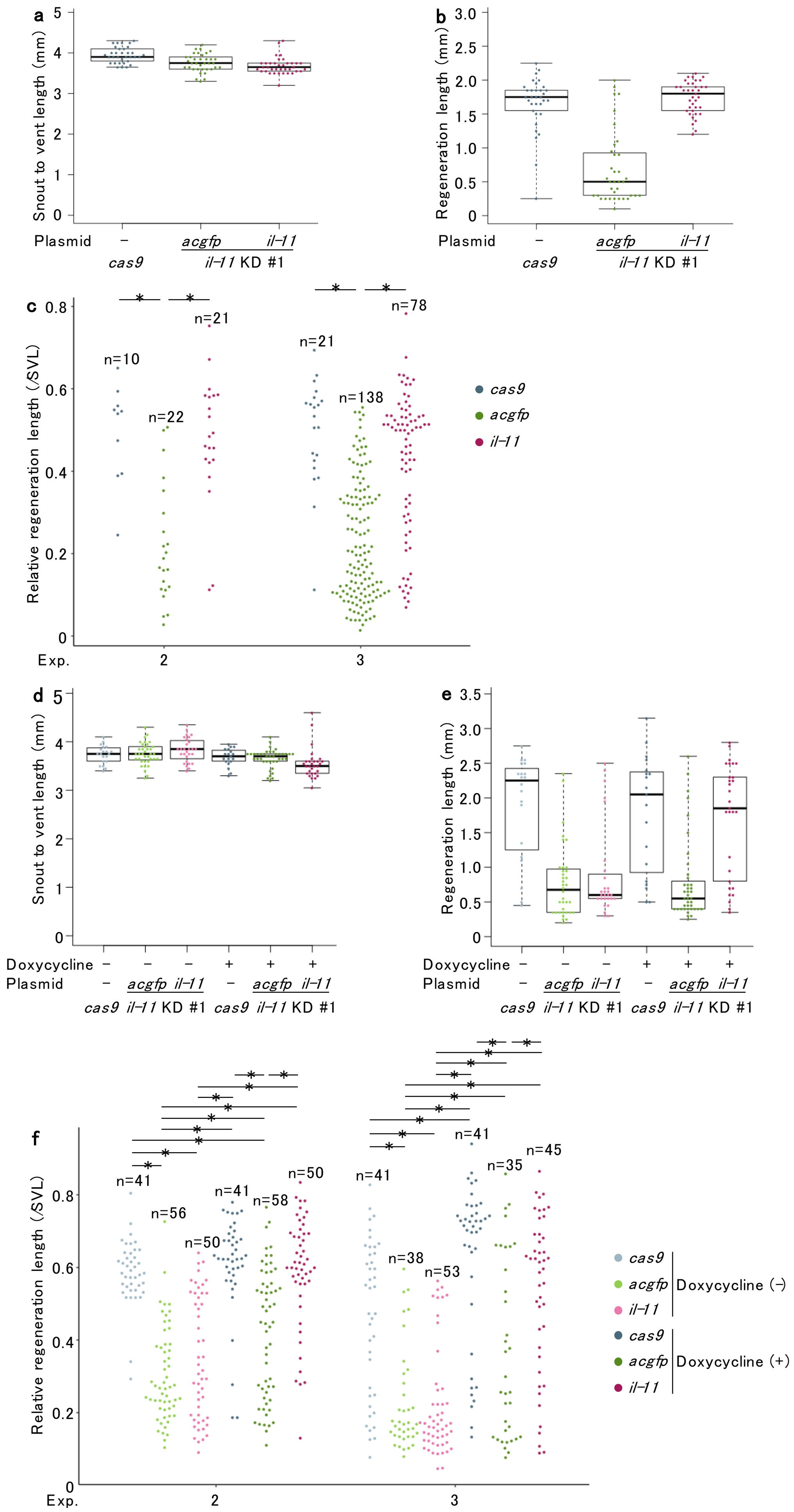
a
Snout to vent length (mm)
Plasmid
–
cas9
acgfp
il-11
il-11 KD #1
b
Regeneration length (mm)
c
Relative regeneration length (/SVL)
Exp.
n=10
n=22
n=21
n=21
n=138
n=78
cas9
acgfp
il-11
d
e
Doxycycline
f
n=41
n=56
n=50
n=41
n=58
n=50
n=41
n=38
n=53
n=41
n=35
n=45
Doxycycline (−)
Doxycycline (+)

Supplementary Figure 5 Analyses of *il-11* forced expressed *il-11* knocked-down tadpoles

a, **b**, **d, e,** Snout to vent length (SVL, a, d) and regeneration length (b, e) used for calculation in Fig. 3j (a, b) or Fig. 3x (d, e) are shown. **c**, **f**, Biological replicates for Fig. 3j (c) or Fig. 3x (f) using different batches of tadpoles are shown. Box plots are inserted in the panels. Bars in the boxes represent median, upper and lower limits of the boxes represent the first and third quartiles, and whiskers represent maximum and minimum values. *$P < 0.05$, Tukey-Kramer's test.

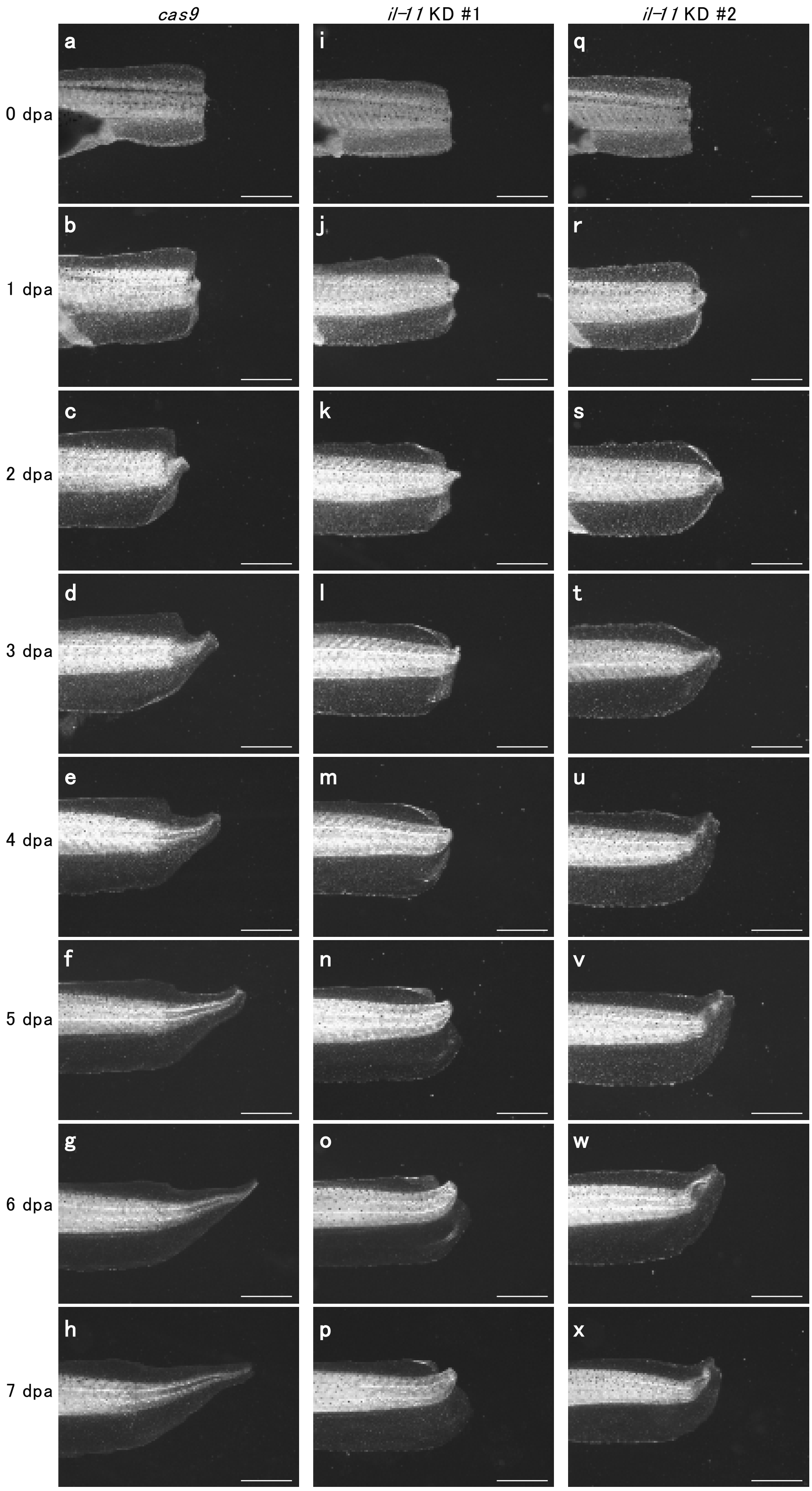

Supplementary Figure 6 The effect of *il-11* knock down becomes prominent between 2 and 3 dpa

Time-lapse images of a *cas9* mRNA-injected (a-h), *il-11* KD #1 (i-p), or #2 (q-x) tadpole are shown. Images were taken at 0 (a, i, q), 1 (b, j, r), 2 (c, k, s), 3 (d, l, t), 4 (e, m, u), 5 (f, n, v), 6 (g, o, w), and 7 (h, p, x) dpa. Anterior is to the left, dorsal is top. Scale bars: 1 mm.

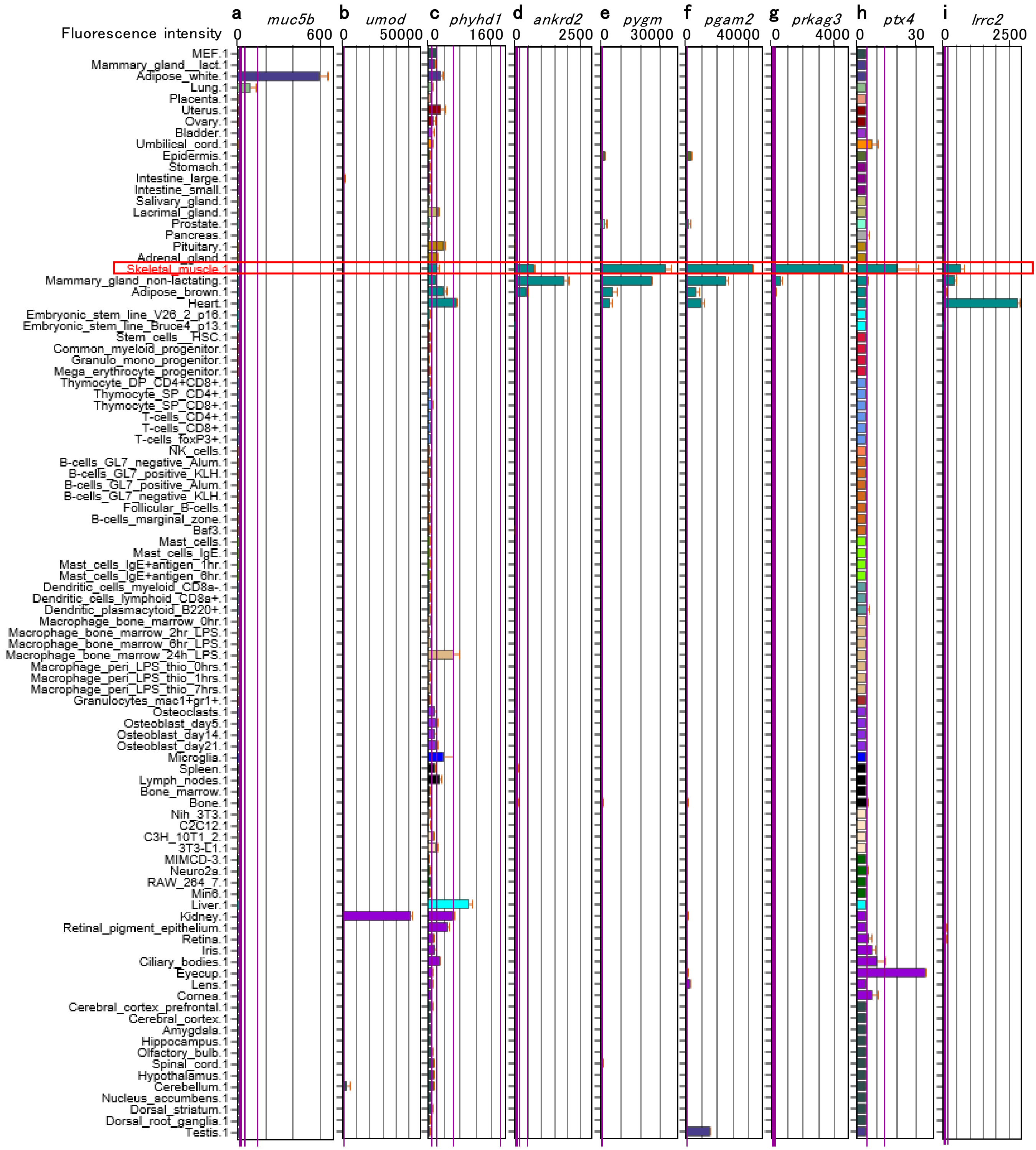

Supplementary Figure 7 Comparison of expression levels of genes upregulated in *il-11* KD #1 tadpoles in different tissues in mice

Expression levels of *mucin 5, subtype B, tracheobronchial* (*muc5b*; a), *uromodulin* (*umod*; b), *phytanoyl-CoA dioxygenase domain containing 1* (*phyhd1*; c), *ankyrin repeat domain 2* (*stretch responsive muscle; ankrd2*; d), *muscle glycogen phosphorylase* (*pygm*; e), *phosphoglycerate mutase 2* (*pgam2*; f), *protein kinase, AMP-activated, gamma 3 non catalytic subunit* (*prkag3*; g), *pentraxin 4* (*ptx4*; h), and *leucine rich repeat containing 2* (*lrrc2*; i) in mouse tissues are shown. Probe sets used were 1427626_at (a), 1456306_a_at (b), 1428394_at (c), 1419621_at (d), 1448602_at (e), 1418373_at (f), 1444480_at (g),

1430194_at (h), or 1427388_at (i). Horizontal axes represent fluorescent intensity. Expression levels in skeletal muscle are indicated in a red box. n=2, Mean ± s.e.m. These data were obtained from BioGPS[1-3] using a microarray data set of mouse tissues in a previous report[4].

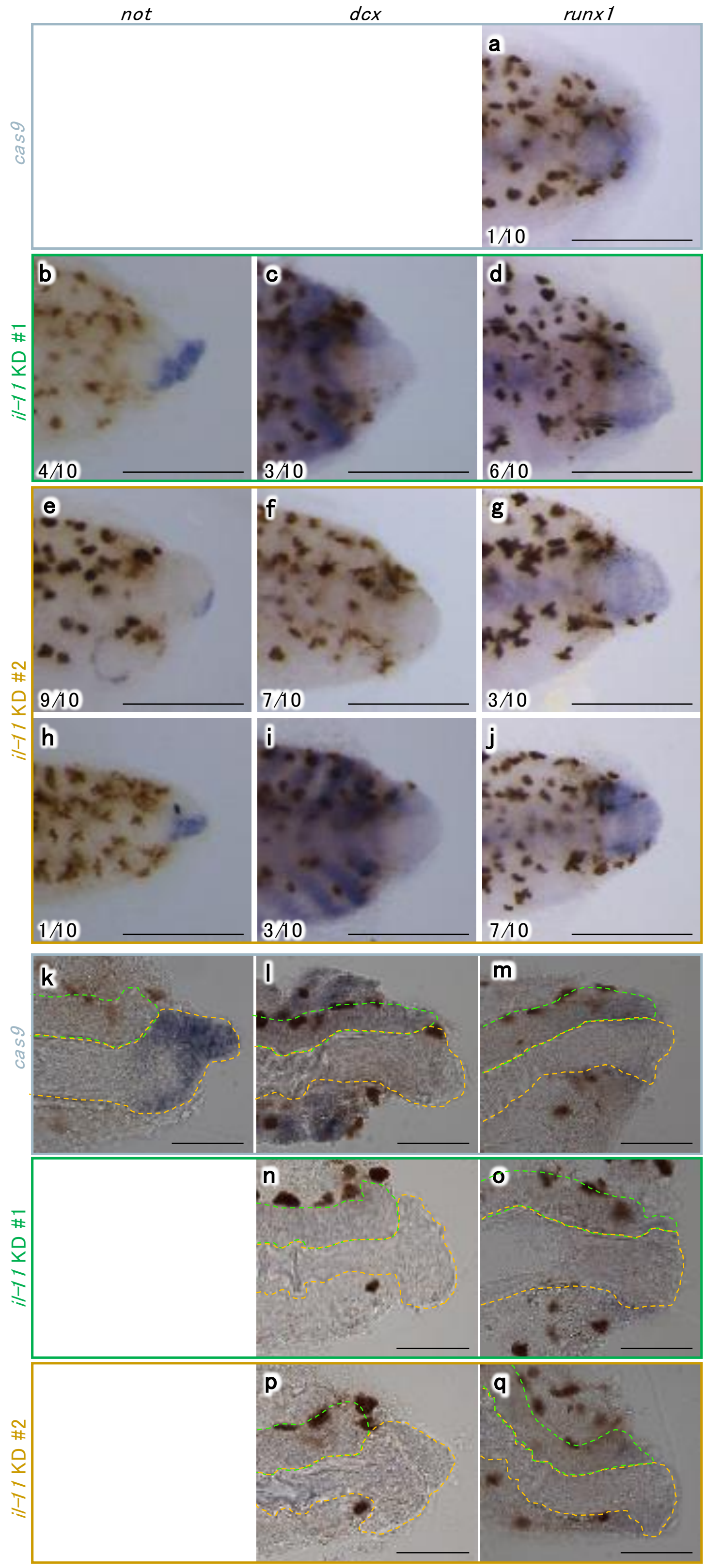

Supplementary Figure 8 Detailed localisation analysis of undifferentiated marker genes in *il-11* KD tadpoles

a-**d**, WISH images for undifferentiated marker genes using *cas9* mRNA-injected (a) or *il-11* KD #1 (b-d) tadpoles that are not shown in Fig. 5. Representative WISH images for *not* (b), *dcx* (c), or *runx1* (a and d) are shown. **e**-**j**, WISH images for undifferentiated marker genes using *il-11* KD #2 tadpoles. Representative

WISH images for *not* (e and h), *dcx* (f and i), or *runx1* (g and j) are shown. **k-q**, Section images. Representative images for *not* (k), *dcx* (l, n and p), or *runx1* (m, o and q) using *cas9* mRNA-injected (k-m), *il-11* KD #1 (n and o), or #2 (p and q) are shown. Anterior is to the left and dorsal is up. Blue/purple colour represents signals for each gene. Brown pigments are melanophores of the tadpoles. Scale bars: 500 μm (a-j) or 100 μm (k-q). Numbers at the bottom corner are the total ratio of tadpoles showing the corresponding expression from two batches. Green broken lines indicate spinal cord and spinal cord ampulla, and yellow broken lines indicate notochord and notochord bud.

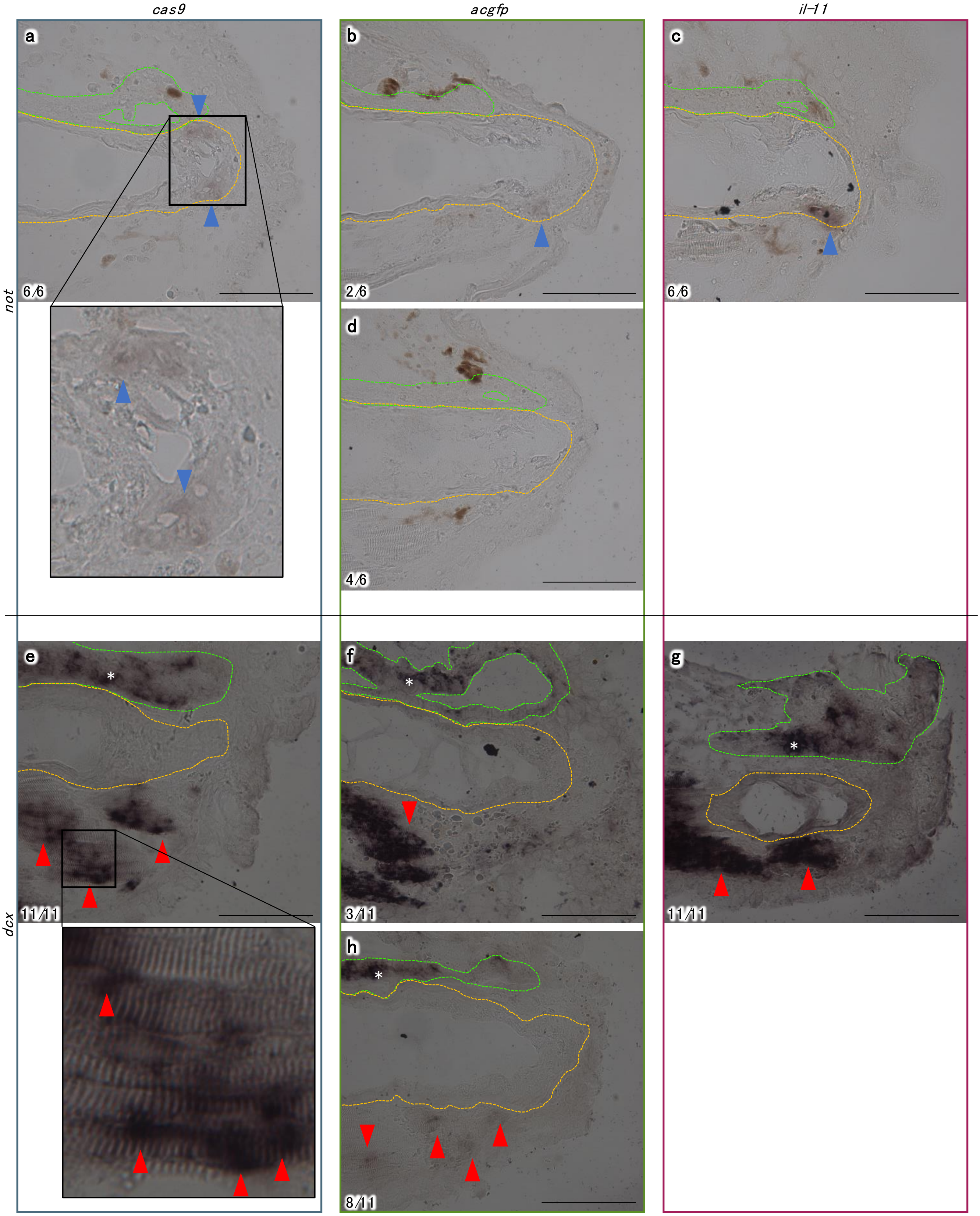

Supplementary Figure 9 Undifferentiated cells are rescued by forced expression of *il-11* in *il-11* KD tadpoles

ISH images for *not* (a-d) or *dcx* (e-h). *cas9* mRNA injected (a, e), *acgfp* (b, d, f, h) or i*l-11* (c, g)-expressing *il-11* KD tadpoles were fixed at 2 dpa, and sagittally sliced. Anterior is to the left, dorsal is up. Scale bars: 100 µm. Magenta/purple colour represents signals for the genes. Brown pigments are melanophores of the

tadpoles. Signals for *not* were detected in the notochord bud (blue arrowheads). Signals for *dcx* were detected in muscle (red arrowheads) and spinal cord (white asterisks). Numbers at the bottom corner are the total ratio of tadpoles, indicating the corresponding expression from at least two batches. Green broken lines indicate spinal cord and spinal cord ampulla, and yellow broken lines indicate notochord and notochord bud. Inset: magnified view of boxed area.

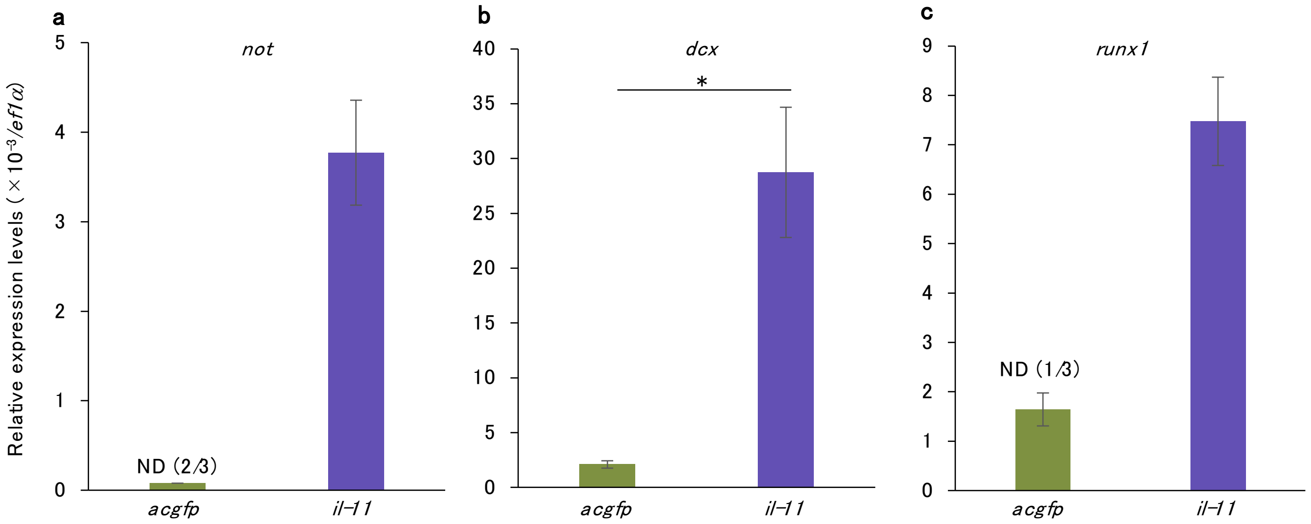

Supplementary Figure 10 Gain of function analysis of *il-11* for expression of undifferentiated markers

Biological replicates for Fig. 6h-j using tadpoles from a different batch. Expression levels of *not* (a), *dcx* (b), or *runx1* (c) were measured by qRT-PCR. Four to six tadpoles were used in each lot. Vertical axes represent relative expression levels normalised by those of *ef1α*. Mean ± s.e.m. n=3. *$P < 0.05$, Student's *t*-test. RT-PCR signal for *not* (a) or *runx1* (c) was not detected in two or one samples in *acgfp*-expressing tails due to low expression levels.

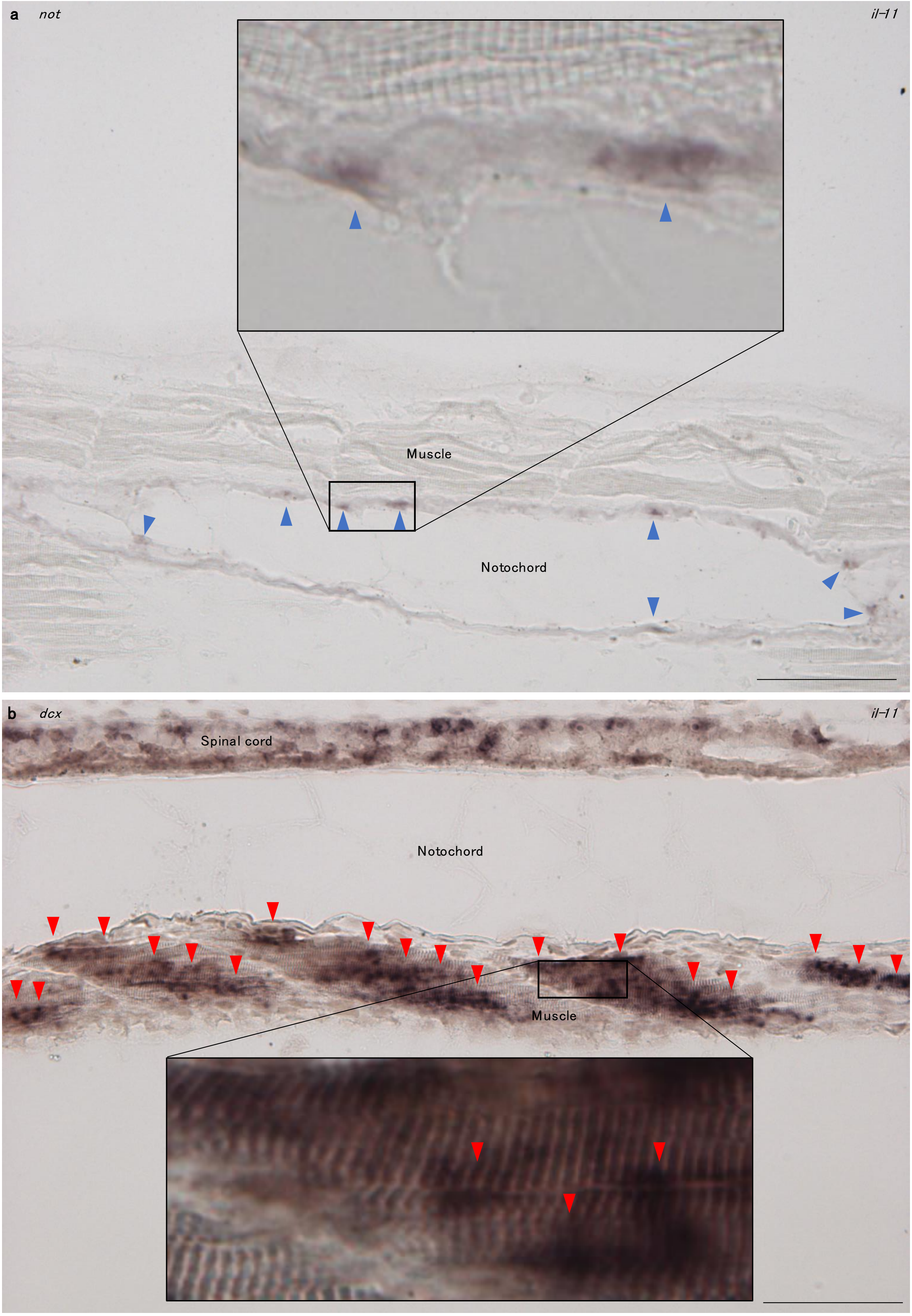

Supplementary Figure 11 Localisation of cells expressing undifferentiated markers in

tadpoles with forced expression of *il-11*

Biological replicates for Fig. 6l and o. Representative ISH images for *not* (a) or *dcx* (b) in *il-11*-expressing intact tadpole tails 2 days after doxycycline treatment are shown. Several *not*-expressing cells in the notochord sheath (blue arrowheads), and *dcx*-expressing muscle (red arrowheads) were detected. Scale bars: 100 μm. Anterior is to the left, dorsal is up. Magenta/purple colour represents signals for the genes. Inset: magnified view of boxed area.

Supplementary Tables

Supplementary Table 1. Survival rate of tadpoles used in KD experiments

Exp.	The ratios of normally developed tadpoles at 6 dpf			The ratio of normally grown tadpoles after tail amputation		
	cas9	*il-11* KD #1	*il-11* KD #2	*cas9*	*il-11* KD #1	*il-11* KD #2
**1**	122/165(74%)	129/177(73%)	115/147(78%)	20/20(100%)	19/20(95%)	20/20(100%)
**2**	72/90(80%)	60/90(67%)	NA	20/20(100%)	20/20(100%)	NA
**3**	80/90(89%)	73/90(81%)	74/90(82%)	20/20(100%)	20/20(100%)	20/20(100%)
**4**	42/86(49%)	29/91(32%)	45/89(51%)	20/20(100%)	20/20(100%)	20/20(100%)
**5**	63/90(70%)	96/180(53%)	NA	17/20(85%)	18/20(90%)	NA
**6**	59/77(77%)	99/184(54%)	NA	19/20(95%)	18/20(90%)	NA
**7**	87/158(55%)	138/268(51%)	131/202(65%)	20/20(100%)	18/20(90%)	19/20(95%)
**8**	134/197(68%)	131/201(65%)	127/191(66%)	20/20(100%)	19/20(95%)	19/20(95%)
**9**	125/179(70%)	128/182(70%)	161/185(87%)	20/20(100%)	17/20(85%)	20/20(100%)
**10**	62/141(44%)	85/143(59%)	71/136(52%)	22/22(100%)	19/20(95%)	20/20(100%)
**11**	116/152(76%)	122/184(66%)	101/186(54%)	20/20(100%)	20/20(100%)	20/20(100%)
**12**	105/196(54%)	77/160(48%)	77/160(48%)	20/20(100%)	15/20(75%)	20/20(100%)
**13**	38/172(21%)	47/180(26%)	41/180(23%)	19/19(100%)	19/19(100%)	19/19(100%)

Exp.: experiment number, NA: not analysed.

Supplementary Table 2. Survival rate of tadpoles used in rescue experiments

Exp.	The ratios of normally developed tadpoles at 6 dpf			The ratios of tadpoles selected for the measurement		
	cas9	*acgfp*	*il-11*	*cas9*	*acgfp*	*il-11*
**1**	37/45(82%)	55/157(35%)	65/150(43%)	33/33(100%)	36/55(65%)	37/65(57%)
**2**	12/40(30%)	37/152(24%)	52/216(24%)	10/11(91%)	22/37(59%)	21/52(40%)
**3**	37/45(82%)	175/265(66%)	107/230(47%)	21/21(100%)	138/168(82%)	78/104(75%)

Exp.: experiment number.

Supplementary Table 3. Survival rate of tadpoles used in rescue experiments by forced expression of *il-6*

Exp.	The ratios of normally developed tadpoles at 6 dpf			The ratios of tadpoles selected for the measurement		
	cas9	*acgfp*	*il-6*	*cas9*	*acgfp*	*il-6*
**1**	32/79(41%)	30/135(22%)	43/145(30%)	32/32(100%)	18/30(60%)	31/43(72%)
**2**	18/43(42%)	72/177(41%)	64/179(36%)	18/18(100%)	47/72(65%)	40/64(63%)
**3**	47/107(44%)	39/174(22%)	69/220(31%)	44/47(94%)	31/39(79%)	28/69(41%)
**4**	13/87(15%)	34/198(17%)	33/185(18%)	12/13(92%)	22/34(65%)	16/33(48%)
**5**	31/64(48%)	22/180(12%)	21/179(12%)	31/31(100%)	14/22(64%)	8/21(38%)

Exp.: experiment number.

Supplementary Table 4. Survival rate of tadpoles used in rescue experiments with or without doxycycline treatment

**Exp.**	**The ratios of normally developed tadpoles at 6 dpf**			**The ratio of normally grown tadpoles after tail amputation**					
	cas9	*acgfp*	*il-11*	*cas9*-off	*acgfp*-off	*il-11*-off	*cas9*-on	*acgfp*-on	*il-11*-on
**1**	42/109 (39%)	82/180 (46%)	72/178 (40%)	20/21 (95%)	36/41 (88%)	28/36 (78%)	19/21 (90%)	40/41 (98%)	29/36 (81%)
**2**	82/118 (69%)	120/182 (66%)	105/181 (58%)	41/41 (100%)	56/60 (93%)	50/52 (96%)	41/41 (100%)	58/60 (97%)	50/53 (94%)
**3**	86/179 (48%)	84/180 (47%)	109/176 (62%)	41/43 (95%)	38/42 (90%)	53/54 (98%)	41/43 (95%)	35/42 (83%)	45/55 (82%)

Exp.: experiment number, off: without doxycycline treatment, on: with doxycycline treatment.

Supplementary References

1. Wu, C. *et al.* BioGPS: an extensible and customizable portal for querying and organizing gene annotation resources. *Genome Biol.* **10,** R130 (2009).
2. Wu, C., MacLeod, I. & Su, A. I. BioGPS and MyGene.info: Organizing online, gene-centric information. *Nucleic Acids Res.* **41,** D561–D565 (2013).
3. Wu, C., Jin, X., Tsueng, G., Afrasiabi, C. & Su, A. I. BioGPS: Building your own mash-up of gene annotations and expression profiles. *Nucleic Acids Res.* **44,** D313–D316 (2016).
4. Lattin, J. E. *et al.* Expression analysis of G Protein-Coupled Receptors in mouse macrophages. *Immunome Res.* **4,** 5 (2008).
