# Supplementary material for: interleukin-11 induces and maintains progenitors of different cell lineages during Xenopus tadpole tail regeneration
Source: Nat Commun. 2017 Sep 8;8:495. doi: 10.1038/s41467-017-00594-5 (PMC5591189; doi:10.1038/s41467-017-00594-5)
Supplement: Supplementary file 1 — Peer Review File [file 41467_2017_594_MOESM1_ESM.pdf]

Reviewers' Comments:

Reviewer #1 (Remarks to the Author)

The present manuscript by Tsujioka and coworkers defines a role for the cytokine Interleukin-11 in Xenopus tadpole tail regeneration. The authors build upon their previous finding via RNA-sequencing that IL-11 is expressed in the proliferating cells of the regenerating tadpole tails after amputation (Tsujioka et al. PLoS One. 2015 Mar 16;10(3):e0111655). Here, the authors significantly enhance this finding by showing that (i) knock-down of il-11 reduced tail regeneration, (ii) rescue of this phenotype by overexpression of il-11, and (iii) induction of several different maker genes in dependence of il-11.

Recombinant Interleukin-11 (IL-11) is approved by the FDA under the trade name Neumega as an agent to treat thrombocytopenia following chemotherapy. Furthermore, the receptor for IL-11 (IL-11R) has gained reasonable attention in recent years due to the fact that it is overexpressed in different kinds of human tumors. Consequently, inhibition of IL-11 signaling, either via blocking antibodies or IL-11 muteins, has been demonstrated (at least in rodent models) to have a beneficial effect in these IL-11-driven tumors.

Tsujioka and coworkers now define a completely different role or IL-11 as a key protein for regeneration of a Xenopus laevis tadpoles tail. To me as a non-specialist in development, the data concerning the regeneration phenotype look very convincing. The experiments have been performed with large numbers of animals, and the statistical analyses are appropriate. The techniques for DNA manipulation using CRISPR/Cas9 are state of the art. This is an important contribution which will gain a lot of interest both in the cytokine field as well as in the field working on regeneration and development.

However, I have a number of points concerning the cytokine and signaling part of the manuscript, which the authors should address:

• Figure 1: The authors show a time-course analysis of il-11 RNA expression using qRT-PCR. Although the authors have shown in their previous publication that the only cytokine upregulated after injury is IL-11, it is important to do the same analysis for other member of the cytokine family, at least for IL-6 and LIF, as these two are (at least in mammals) very important in terms of differentiation and maintenance of stem cells, progenitor cells etc.
• Along the same line: What is known about the receptor(s) of IL-11 in this system? Which cells express IL-11R and gp130 and are thus the targets of IL-11? Stainings and/or qRT-PCR should be performed.
• Figure 3: The rescue experiment with overexpression of IL-11 is very convincing, as it completely reverses the knock-out phenotype. The question remains how specific this effect is. Could any other cytokine or factor that is able to activate gp130 and thus Xstat3 rescue this phenotype? If e.g. IL-6 would also be able to rescue this, it would not necessarily weaken the manuscript, but would provide additional insight into the signaling pathways underlying the observed phenotype.
• Did the authors check for Xstat3 activation? This appears to be the major transcription factor downstream of IL-11, and would be a good candidate to drive the cell proliferation and/or progenitor maintenance in this model. Stainings to visualize Xstat3 activation would be very helpful.
• The manuscript is rather short and very condensed. In my opinion the manuscript would benefit from more explanation of the impressive results, which would it make also more easily accessible for the interest reader.

Reviewer #2 (Remarks to the Author)

This is a very nice manuscript that analyzes the role of IL11 in Xenopus tail regeneration using CRISPR-mediated gene knockout, combined with insertion of a rescue cassette. IL11 is expressed in the regeneration blastema and knockout of IL11 in F0 Xenopus tadpoles leads to apparently normal development, but defective tail regeneration. Integration of a tet-inducible rescue construct together the KO gRNAs yields rescue of the regeneration defect. Overexpression of IL11 in intact tadpoles causes an upregulation of some genes associated with the blastema, as detected by PCR.

In general, this is an excellent contribution that uses CRISPR KO and rescue to analyze the role of this immune regulator in regeneration. The numbers of animals analyzed are impressive. However, there are a number of places where it needs to be clarified exactly how the experiment was done in order to assess the conclusiveness of the data. In addition, some of the expression localization needs to be performed at higher resolution in order to merit the text used to describe the conclusions.

1. In the rescue experiment in which a tet-inducible IL11 expression construct is knocked in to animals simultaneous with IL11 knockout. The control is termed “mock”. It is unclear if the “mock” control are parallel animals that were never induced, or whether the “mock” control are the same set of animals that were amputated first with no induction and then subsequently amputated again with induction. Considering that the authors are using a heterogeneous set of F0 animals, it is important for the authors to perform the control experiment and the rescue experiment on the same animals—once without induction and once with induction to really confirm that there is a regeneration defect in the animals that can be rescued by expression of IL11.

2. Throughout the manuscript the authors discuss the effect of the IL11 perturbations on progenitor cells. However, none of the data are presented at the resolution of a cell. All of the in situs are whole mount in situs that have tissue level resolution. The in situ analysis of downstream genes in the WT, KO and the KO/rescue needs to be performed at higher resolution—cross sections with in situ hybridization.

3. The authors also show that induction of IL11 in the mature tissue causes upregulation of certain downstream targets by PCR. However, this data states that the mock (WT) expression is Zero while the relative expression after induction is around 1. This numerical system is difficult to understand, as anything relative to 0 would be infinity. In any case, the expression pattern of the induction would need to be confirmed by in situ hybridization at the cellular level. This is particularly important because in figure 5d-g it seems that the F0 transgenics are mosaically expressing the expression construct in different tissues, depending on the individual. In the text, (line 144) the authors conclude that IL11 is “sufficient to induce and maintain progenitor cells even in intact tailssufficient to induce and maintain progenitor cells even in intact tails”. I would say that analysis of three markers by PCR is not sufficient to conclude that progenitor cells are induced in such an experiment. Rather IL11 is upstream of these marker expression.

Reviewer #1:

• Figure 1: The authors show a time-course analysis of il-11 RNA expression using qRT-PCR. Although the authors have shown in their previous publication that the only cytokine upregulated after injury is IL-11, it is important to do the same analysis for other member of the cytokine family, at least for IL-6 and LIF, as these two are (at least in mammals) very important in terms of differentiation and maintenance of stem cells, progenitor cells etc.

All of the comments of Reviewer #1 are very constructive to provide additional insights to understand the role of IL-11 signaling in tail regeneration.

To address the first comment, we performed qRT-PCR for *il-6* and *lif* (Supplementary Figs. 2a-d), as suggested by Reviewer #1, and found that the expression of both of them were not prominently elevated (at most less than ~3-folds) compared to that of *il-11* after tail amputation, suggesting that *il-11* has a special role in tail regeneration among IL-6 family members. We explained these results in the revised manuscript (p. 4, ls. 88-93).

• Along the same line: What is known about the receptor(s) of IL-11 in this system? Which cells express IL-11R and gp130 and are thus the targets of IL-11? Stainings and/or qRT-PCR should be performed.

Thank you for these important comments. According to the Reviewer #1's comment, we have added some sentences to explain about the IL-11 receptors in the Introduction section on p. 2, ls. 37-44.

We have performed qRT-PCR and WISH for *il11ra* (*il-11r*) and *il6st* (*gp130*), according to the Reviewer #1's comment. qRT-PCR analysis revealed that both of them were not prominently upregulated (at most less than ~3-folds) after tail amputation (Supplementary Figs. 2e-h), suggesting that the cells responsive to IL-11 constitutively reside in intact tadpole tails and the number of the cells remains largely unaltered during the regeneration process. We explained this results on p. 5, ls. 103-108.

Although identification of cells expressing the receptors is an important question as Reviewer #1 pointed out, WISH signals for *il11ra* and *il6st* were not detected in the present system, possibly due to their low expression levels. It is necessary for us to improve the sensitivity of the WISH system to identify the cells

expressing the receptors in our future study.

• Figure 3: The rescue experiment with overexpression of IL-11 is very convincing, as it completely reverses the knock-out phenotype. The question remains how specific this effect is. Could any other cytokine or factor that is able to activate gp130 and thus Xstat3 rescue this phenotype? If e.g. IL-6 would also be able to rescue this, it would not necessarily weaken the manuscript, but would provide additional insight into the signaling pathways underlying the observed phenotype.

Thank you for this important comment. According to the Reviewer #1's comment, we designed a construct for the forced expression of *il-6*, and examined whether forced expression of *il-6* rescues the phenotype of *il-11* KD tadpoles to answer the reviewer's question. We found that the average regeneration length of *il-6* forced expressed tadpoles was significantly shorter than that of the control tadpoles (Supplementary Fig.6), suggesting that *il-11* selective pathway is necessary for tail regeneration. We explained this on p. 7, ls. 163-169.

• Did the authors check for Xstat3 activation? This appears to be the major transcription factor downstream of IL-11, and would be a good candidate to drive the cell proliferation and/or progenitor maintenance in this model. Stainings to visualize Xstat3 activation would be very helpful.

Thank you for this important comment. We performed immunohistochemistry using the antibody against phosphorylated Stat3 to visualize activation of Stat3 at 72 hpa and found that phosphorylated Stat3 localized in cells in notochord bud, spinal cord ampulla, and epithelium in blastema (Supplementary Fig. 3). Although Stat3 is activated by several upstream ligands including IL-6 and LIF but these two ligands were not prominently elevated compared to *il-11* after tail amputation, as mentioned above, thus it is plausible that the observed activation of Stat3 is mainly due to the expression of IL-11 and that IL-11 signaling is activated in various tissues in blastema. We explained this result on p. 4, l. 94- p. 5, l. 102.

• The manuscript is rather short and very condensed. In my opinion the manuscript would benefit from more explanation of the impressive results, which would it make also more easily accessible for the interest reader.

Thank you for this helpful comment. According to the Reviewer #1's comment, we have added (1) information about the IL-11 signaling pathways on the introduction (p. 2, l. 45- p.3, l. 55) in order to make it easy for non-specialist of cytokines to interpret the results, (2) data about mortality rate of the injected tadpoles (Supplementary Table 1-4, ps. 6, 8, ls. 126-131, 175-178), because the information would be helpful for specialists using *Xenopus*, (3) explanation of the rescue and forced expression experiment systems (ps. 6, 7, 10, 15, 17, 18, ls. 143-146, 148-150, 153-158, 234-238, 373-375, 400-401, 411-413, 437-439) to clarify our experimental design, and (4) discussions about some results (p. 11, l. 259-p. 12, l. 294) to provide additional insights into the results. We also changed the way to display the result of KD/rescue. We used beeswarm plot combined with box plot instead of bar graph to provide more information of heterogenous distribution of the results for F_0 animals (Figs. 2, 3, Supplementary Figs. 4, 5). We also pointed out that the brown pigments in WISH/ISH images are melanophores, because some readers might misunderstand that the brown color is the signal (ps. 26, 32, 35, 50, ls. 584-585, 645, 680-681, 762).

Reviewer #2:

1. In the rescue experiment in which a tet-inducible IL11 expression construct is knocked in to animals simultaneous with IL11 knockout. The control is termed "mock". It is unclear if the "mock" control are parallel animals that were never induced, or whether the "mock" control are the same set of animals that were amputated first with no induction and then subsequently amputated again with induction. Considering that the authors are using a heterogeneous set of F0 animals, it is important for the authors to perform the control experiment and the rescue experiment on the same animals—once without induction and once with induction to really confirm that there is a regeneration defect in the animals that can be rescued by expression of IL11.

We agree that this comment is very important. First, the 'mock' control indicated 'tadpoles in which *acgfp*-expressing construct but not *il-11*-expressing construct had been introduced and then *acgfp* expression was forced by doxycycline-treatment' in our previous version. Therefore, it did not mean either 'parallel animals that were never induced' or 'the same set of animals that were amputated first with no induction and then subsequently amputated again with induction', as Reviewer #2 supposed in his/her

comment. We are sorry for our shortage in explanation.

We agree that the proposed experiment is completely reasonable, when one considers that we are using a heterogeneous set of F_0 animals. The nature of the animal we use, however, makes it very difficult to perform the exact experiment that Reviewer #2 proposed. *X. laevis* tadpoles loose the tail regenerative ability during certain developmental stages (in our condition, approximately 7-28 dpf), called 'refractory period' (Slack et al., Molecular Pathways Needed for Regeneration of Spinal Cord and Muscle in a Vertebrate. Dev. Cell, 5, 429-439, 2003). Therefore, the only way to perform the proposed experiment is to amputate tails of tadpoles once during 'pre-refractory regeneration capable period (5, 6 dpf)', and once more during 'post-refractory regeneration capable period (28 dpf or later)'. We are afraid, however, that the interpretation of the results may become quite difficult, because in addition to the body size (the body length of the post-refractory period tadpoles is more than 3 times longer than that of pre-refractory period tadpoles), the speed of tail regeneration may considerably differ between these two distinct developmental stages.

Therefore we designed an alternative experiment to address the reviewer's concern that regeneration defect in *il-11* KD tadpoles are really rescued by the expression of IL-11. We created KD/rescue tadpoles as described in our previous manuscript, and divided them into two groups: tadpoles in the first group are treated with doxycycline, whereas tadpoles in the second group are untreated. In this sense, the "mock" control in our revised version are 'parallel animals that were forced to express *acgfp*', although they are not 'pararell animals that were never induced'. We thought, however, by testing enough number of animals, we can safely conclude whether the regeneration defects in *il-11* KD tadpoles are really rescued by the expression of IL-11.

Using this experimental design, we examined whether the rescued phenotype is dependent on doxycycline treatment, which is thought to correlate with the expression of IL-11.We found that regeneration length of *il-11* expressing construct-introduced *il-11* KD tadpoles was almost as short as that of *acgfp* expressing construct-introduced *il-11* KD tadpoles <u>in untreated group</u>, whereas it was almost as long as that of control *cas9* mRNA-injected tadpoles <u>in doxycycline treated group</u> (Figs. 3l-x). An enough number (n=19 or more) of animals were used in this experiment, and this experiment was well reproduced (Supplementary Fig. 5f). Therefore, although the same animals are not compared, we expect that this result would well address Reviewer #2's concern that the regeneration defect in *il-11* KD tadpoles that we reported in our previous version was due to mere experimental variation. We explained this experiment on p.7, l. 170-p. 8, l. 189.

To clarify that control construct is *acgfp*-expressing construct, we also changed the term “mock” to “*acgfp*”.

2. Throughout the manuscript the authors discuss the effect of the IL11 perturbations on progenitor cells. However, none of the data are presented at the resolution of a cell. All of the in situs are whole mount in situs that have tissue level resolution. The in situ analysis of downstream genes in the WT, KO and the KO/rescue needs to be performed at higher resolution—cross sections with in situ hybridization.

Thank you very much for this very important comment. According to the Reviewer #2’s comment, we performed section ISH for the marker genes using (1) *cas9*-mRNA injected, (2) *acgfp*, or (3) *il-11* expressing *il-11* KD tadpoles, which we suppose to correspond to (1) WT, (2) KO, and (3) KO/rescue tadpoles, respectively. Although we could not detect reliable signals for *runx1* due to high background in our experimental system, we detected signals for *not* and *dcx* (Supplementary Fig. 10). We found *not* was expressed in cells in notochord and *dcx* was expressed in muscle, and downregulation of them in *il-11* KD tadpoles were rescued by forced expression of *il-11*. Although reliable signals for *runx1* was not detected, we think these results strongly suggest that *il-11* induces and maintains progenitor cells of different lineages in tail regeneration. These results are explained on p. 9, l. 221-p. 10, l. 230.

3. The authors also show that induction of IL11 in the mature tissue causes upregulation of certain downstream targets by PCR. However, this data states that the mock (WT) expression is Zero while the relative expression after induction is around 1. This numerical system is difficult to understand, as anything relative to 0 would be infinity. In any case, the expression pattern of the induction would need to be confirmed by in situ hybridization at the cellular level. This is particularly important because in figure 5d-g it seems that the F0 transgenics are mosaically expressing the expression construct in different tissues, depending on the individual. In the text, (line 144) the authors conclude that IL11 is “sufficient to induce and maintain progenitor cells even in intact tails sufficient to induce and maintain progenitor cells even in intact tails”. I would say that analysis of three markers by PCR is not sufficient to conclude that progenitor cells are induced in such an experiment. Rather IL11 is upstream of these marker

expression.

We agree that the scales of the qRT-PCR were confusing, and changed them. Vertical axes in the revised manuscripts now indicate the relative expression levels of the genes normalized by that of *ef1a* (Figs. 1a, b, 5h-j, Supplementary Figs. 2, 11).

We agree that ISH is necessary to conclude that progenitor cells are induced by forced expression of *il-11*. Although reliable signals for *runx1* was not detected due to high background, we detected signals for *not*, and *dcx*, and found that the numbers of cells expressing these marker genes increased in *il-11* expressing cells (Figs. 5k-p), suggesting that *il-11* is sufficient to induce at least *not* and *dcx* expressing progenitor cells. These results are explained on p. 10, l. 246-p. 11, l. 256.

We have also added information about the method used in additional experiments on ps. 13, 14, 16-19, ls. 323-327, 335-34, 395-397, 406-410, 440-451.

Second, we changed the term "*dcx-like*" to "*dcx*", according to the renewal of nomenclature on Xenbase. We modified explanation of dcx accordingly on p. 9, l. 209.

Third, we found a mistake about the statistical method: statistical test used in Supplementary Fig. 4h was not "Tukey-Kramer's", but "Dunnett's" test. Therefore we corrected it on p. 40, l. 711. We sincerely apologize for this mistake.

Forth, we changed the term "N" to "n", to clarify that the term indicates the size of the sample we used.

Fifth, because some of the scale bars were too short, we changed the length of them (Figs. 2f-h, 3d-i, 5d-g, Supplementary Fig. 7). We also changed some of the order of the labelling of the figures to make it more easily accessible for the readers (Figs. 3d-i, 5d-g).

Sixth, we corrected some misspelling in our previous manuscripts.

Seventh, we changed some of the legend titles to clarify the meaning of the figures (ps. 33, 42, ls. 650, 713).

Eighth, we divided results section into some subsections.

We think that the revised version is much more improved than the previous one. We hope that the revised version is now suitable for publication in *Nature Communications*. I am looking forward to hearing from you again.

Reviewers' Comments:

Reviewer #1:
Remarks to the Author:
The authors have performed several new experiments to address my questions and concerns. They have furthermore expanded their manuscript and explain their results in more depth and detail. Overall, the authors have significantly improved their manuscript, which I highly recommend for publication in Nature Communications.

Reviewer #2:
Remarks to the Author:
The manuscript from Tsujioka is significantly improved, especially in the point of proper controls of uninduced animals. The authors have clearly made a sincere effort to address the reviewers' points. There are still a few points in which the manuscript should be improved. This is important to bring the data quality and writing up to a level that will give this impressive study its due respect in the field, which it deserves.

1. Line 99-104. The authors describe the data in Supp Figure 2 as showing that lif and il6st are not significantly induced—and accurately say that not more than 3 –fold. The writing is a matter of interpretation—based on the error bars, one would say there is induction, just not as massive as for Il11. Therefore I would recommend to modify the text to say that lif and il6st are modestly induced compared to IL11.

2. The authors are commended for performing the P-stat3 immunofluorescence and the section in situ hybridizations. However the data/image quality do need to be improved to come up to par with good regeneration publications. The P-stat3 staining looks convincing but needs to be taken at higher magnification. For example, higher mag insets can be one option. Generally, in imaging regenerating sections, people routinely use microscopes that can stitch images together in order to have high resolution over large areas. In the section in situ data, the sections look under fixed, and the section thickness may be too high so cellular resolution is not apparent and the signal does not look convincing. Rather than Bouin's fixative, 4% PFA in PBS or MEMFA are standard. Also, better microscopy conditions with higher magnification are required to obtain sufficient cellular resolution.

3. The data in supplementary figure 8 are not sufficiently explained. It seems that the authors are comparing the genes they found upregulated with a mouse dataset that surveyed multiple tissues. But it is unclear if the histograms presented represent the frog or the mouse data.

Referee #2

The manuscript from Tsujioka is significantly improved, especially in the point of proper controls of uninduced animals. The authors have clearly made a sincere effort to address the reviewers' points. There are still a few points in which the manuscript should be improved. This is important to bring the data quality and writing up to a level that will give this impressive study its due respect in the field, which it deserves.

>1. Line 99-104. The authors describe the data in Supp Figure 2 as showing that lif and il6st are not significantly induced—and accurately say that not more than 3 –fold. The writing is a matter of interpretation—based on the error bars, one would say there is induction, just not as massive as for Il11. Therefore I would recommend to modify the text to say that lif and il6st are modestly induced compared to IL11.

Thank you for this thoughtful comment. We agree that it is more appropriate to say that lif and il6st are modestly induced compared to il-11, and have corrected the manuscript, accordingly.

>2. The authors are commended for performing the P-stat3 immunofluorescence and the section in situ hybridizations. However the data/image quality do need to be improved to come up to par with good regeneration publications. The P-stat3 staining looks convincing but needs to be taken at higher magnification. For example, higher mag insets can be one option. Generally, in imaging regenerating sections, people routinely use microscopes that can stitch images together in order to have high resolution over large areas. In the section in situ data, the sections look under fixed, and the section thickness may be too high so cellular resolution is not apparent and the signal does not look convincing. Rather than Bouin's fixative, 4% PFA in PBS or MEMFA are standard. Also, better microscopy conditions with higher magnification are required to obtain sufficient cellular resolution.

First, we apologize for not having submitted high quality images in the revised manuscript. We submitted low quality images, because file sizes of the original high quality images were too big to be incorporated into the main text. We would like to submit the original images to show the quality of the presumptive final version of the figures. We think that the quality of the original images is high enough, but some modifications would make the paper more easily accessible for the readers. Therefore, we will use higher mag inset for P-Stat3 staining in Supplementary Fig. 3 and in situ hybridization in Supplementary Fig. 10, according to the reviewer's suggestion. We will also show other larger in situ hybridization images for Fig. 5 in a new supplementary figure, because images of Figs. 5k, l, n, o might be too small.

Because the size of most cells of vertebrate is approximately 10 micro meter, 8 micro meter thick section we made in section in situ hybridization is thin enough to reach cell level resolution. Moreover, we chose the appropriate fixative for in situ hybridization: we used Bouin's fixative for notochord homeobox (not), and MEMFA fixative for doublecortin (dcx) as explained in "Tissue section ISH" in "Method". We found that Bouin's fixative is better to preserve the morphology of the section than MEMFA fixative, and used Bouin's fixative for not. Bouin's fixative was not appropriate, however, for dcx, because Bouin's fixative stained muscle pale brown where we found signals for dcx (although these staining can be easily distinguished from in situ signals). Therefore we used MEMFA's fixative instead of Bouin's fixative for dcx. We used the highest power objective lens (x40) we could use, and original images are sufficient for cellular resolution. We think that these images address the concerns reviewer #2 expressed.

>3. The data in supplementary figure 8 are not sufficiently explained. It seems that the authors are comparing the genes they found upregulated with a mouse dataset that surveyed multiple tissues. But it is unclear if the histograms presented represent the frog or the mouse data.

Thank you for this important comment. The histograms represent the mouse data, not the frog data. We have explained this clearly in the revised manuscript.
